# Nanowire FET Based Neural Element for Robotic Tactile Sensing Skin

**DOI:** 10.3389/fnins.2017.00501

**Published:** 2017-09-20

**Authors:** William Taube Navaraj, Carlos García Núñez, Dhayalan Shakthivel, Vincenzo Vinciguerra, Fabrice Labeau, Duncan H. Gregory, Ravinder Dahiya

**Affiliations:** ^1^Bendable Electronics and Sensing Technologies Group, School of Engineering, University of Glasgow Glasgow, United Kingdom; ^2^ST Microelectronics Catania, Italy; ^3^Department of Electrical and Computer Engineering, McGill University Montreal, QC, Canada; ^4^WestCHEM, School of Chemistry, University of Glasgow Glasgow, United Kingdom

**Keywords:** silicon nanowire, tactile skin, sparse coding, Nanowire Field Effect Transistor, neuro-robotics

## Abstract

This paper presents novel Neural Nanowire Field Effect Transistors (υ-NWFETs) based hardware-implementable neural network (HNN) approach for tactile data processing in electronic skin (e-skin). The viability of Si nanowires (NWs) as the active material for υ-NWFETs in HNN is explored through modeling and demonstrated by fabricating the first device. Using υ-NWFETs to realize HNNs is an interesting approach as by printing NWs on large area flexible substrates it will be possible to develop a bendable tactile skin with distributed neural elements (for local data processing, as in biological skin) in the backplane. The modeling and simulation of υ-NWFET based devices show that the overlapping areas between individual gates and the floating gate determines the initial synaptic weights of the neural network - thus validating the working of υ-NWFETs as the building block for HNN. The simulation has been further extended to υ-NWFET based circuits and neuronal computation system and this has been validated by interfacing it with a transparent tactile skin prototype (comprising of 6 × 6 ITO based capacitive tactile sensors array) integrated on the palm of a 3D printed robotic hand. In this regard, a tactile data coding system is presented to detect touch gesture and the direction of touch. Following these simulation studies, a four-gated υ-NWFET is fabricated with Pt/Ti metal stack for gates, source and drain, Ni floating gate, and Al_2_O_3_ high-k dielectric layer. The current-voltage characteristics of fabricated υ-NWFET devices confirm the dependence of turn-off voltages on the (synaptic) weight of each gate. The presented υ-NWFET approach is promising for a neuro-robotic tactile sensory system with distributed computing as well as numerous futuristic applications such as prosthetics, and electroceuticals.

## Introduction: neuro-mimicking tactile sensing

**Figure 1:**
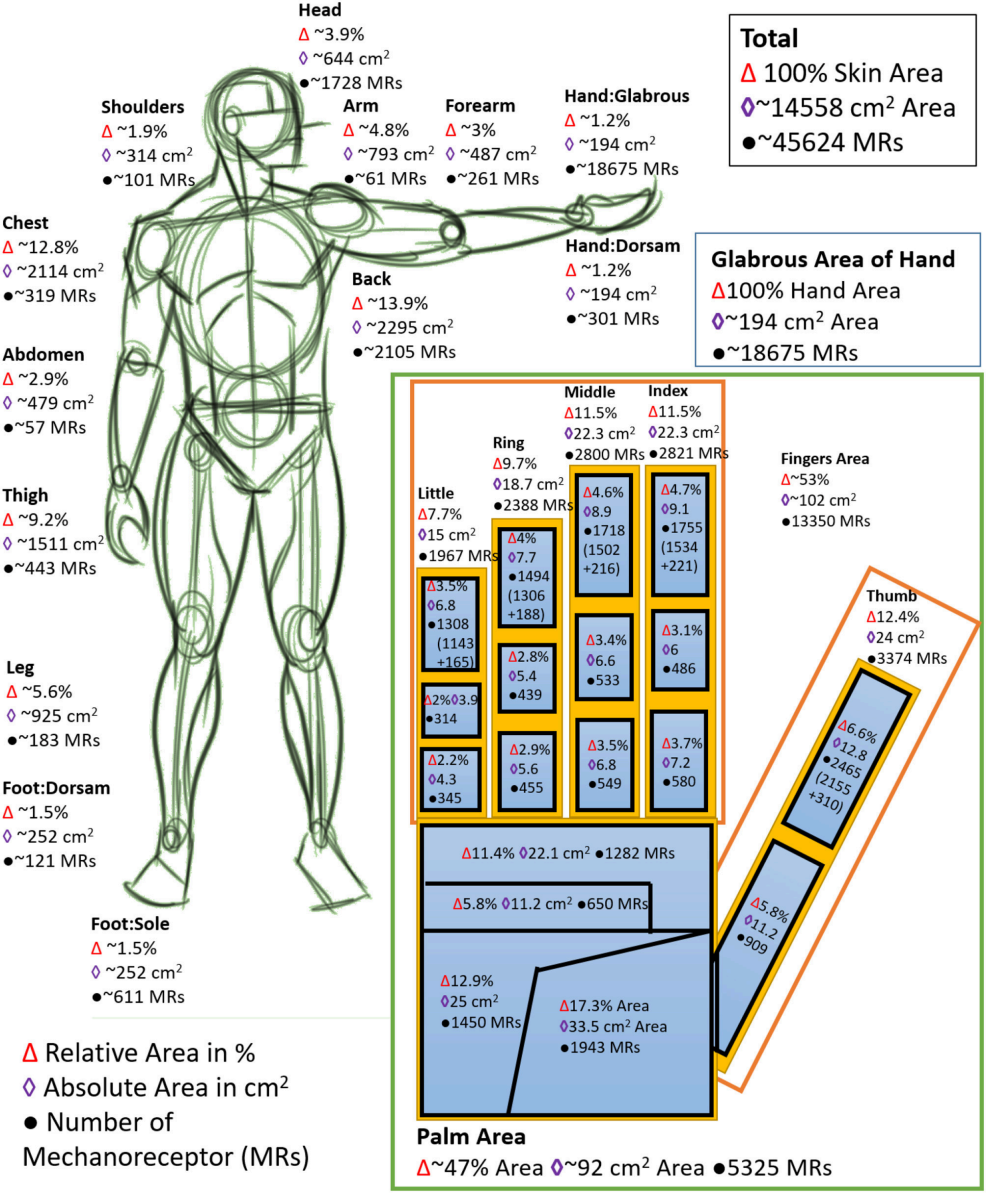
The distribution of mechanoreceptors in various parts of human body. (Inset: 100% glabrous area of hand, which corresponds to 1.2% of the total skin area). The number of mechanoreceptors indicate the typical number of sensors required in various parts to achieve a full body touch/pressure sensing to mimic human body.

Humans and other biological organisms use tactile feedback to interact with the environment (Dahiya et al., 2010). Inspired by nature, numerous research groups are harnessing the technological advances to develop artificial e-skin with features mimicking human skin (Boland, 2010; Tee et al., 2012; Bauer, 2013; Hammock et al., 2013; Wang et al., 2013; Yogeswaran et al., 2015; Núñez et al., 2017). These works find application in prosthetics, potentially to bestow lost sensory feelings to amputees (Raspopovic et al., 2014) and robotics to provide the touch sensory capability allowing them to interact physically and safely with real-world objects (Dahiya et al., 2013b). Thus far, the major focus of e-skin research has been on the development of various types of sensors (e.g., contact pressure, temperature, humidity, etc.) and their integration on large-area and flexible/conformable substrates (Dahiya and Valle, 2013; Dahiya et al., 2013a, 2015; Hammock et al., 2013; Lee et al., 2015; Yogeswaran et al., 2015; Polishchuk et al., 2016; Núñez et al., 2017). However, processing of a large amount of data from e-skin has remained a challenge, especially in the case of large area skin where number of touch sensors increase rapidly. As an example, to develop human inspired e-skin for robotic and prosthetic limbs, an estimated 45 K mechanoreceptors (MRs) will be needed in about 1.5 m^2^ area, as shown in Figure 1 (details in Supplementary Section 1; Johansson and Vallbo, 1979; Boniol et al., 2008; Mancini et al., 2014; Goldstein and Brockmole, 2016). This number of sensors will be much higher if we consider e-skin to have equivalents of thermo-receptors and nociceptors (Goldstein and Brockmole, 2016). The large number of receptors in the skin indicates that the tactile data will multiply rapidly, and therefore one can understand the challenge associated with its compiling and processing. With the recent shift in the focus of tactile skin research in robotics from hands to whole-body tactile feedback, a need has arisen for new techniques to manage the tactile data. Currently, limited solutions are available to deal with large data generated in tactile skin, let alone for the resulting touch based perception, which is another dimension of tactile data handling. For example, in the case of prosthesis, it is important not only to collect the tactile data for critical feedback, but also to decode the user's intentions in real time (Raspopovic et al., 2014). Perhaps a neuron-like inference to handle the tactile data early on could help as indicated by a significant downstream reduction in the numbers of neurons transmitting stimuli in the early sensory pathways in humans (Barlow, 1981; Buck, 1996; Barranca et al., 2014). Research suggests that distributed computing takes place in the biological tactile sensory system (Barlow, 1981; Dahiya et al., 2010, 2015). For example, the ensemble of tactile data from peripheral neurons is considered to indicate both the contact force and its direction (Johansson and Birznieks, 2004; Johansson and Flanagan, 2009). Such distributed local processing of tactile data is advantageous in practical terms as sending reduced data to higher-perceptual level releases some pressure in terms of complex and bulky sensory hardware. Thus, the hardware implemented neuromorphic tactile data processing along with neural networks like algorithms will be helpful. Currently, the neuromorphic hardware is primarily targeted for vision and hearing related applications. Since, vision and hearing are not as distributed as tactile sensing, the neuromorphic hardware developed for them is not optimal for tactile sensing and dedicated solutions are needed. Few works on tactile sensing have used software based neural networks approaches for tasks such as object recognition via texture or materials (Decherchi et al., 2011; Kaboli et al., 2015). However, due to the lack of large-scale parallel processing, the software-programmed neural networks are slower and less energy-efficient (Ananthanarayanan et al., 2009; Misra and Saha, 2010) and hence the HNN implementations will be interesting.

The hardware neuromorphic architecture implementations reported in literature thus far are based on devices such as memistor (Widrow and Hoff, 1960), spin-logic (Sengupta et al., 2015; Grollier et al., 2016), memristor (Jo et al., 2010), neuron MOSFET (Ishii et al., 1992; Kotani et al., 1998), analog circuit based neurons (Mead and Ismail, 2012), field programmable gate array (FPGA) (Misra and Saha, 2010) and software-programmed neural networks (Cotton and Wilamowski, 2010). So far, these technologies have not been used with tactile skin. But, they could offer alternative to the υ-NWFET approach presented here—even if υ-NWFET has many inherent advantages such as possibility of printing devices on large area as discussed in the next section. The above alternative technologies have their own advantages and challenges in terms of complexity, scalability, speed, reliability, repeatability, cost, non-bendability, power consumption etc., which limit their use in the emulation of biological systems. For example, the memistor, a 3-terminal electrochemical cell element achieved limited success because of scalability issues (Widrow and Hoff, 1960). Likewise, the spintronic neurons are energy efficient (Grollier et al., 2016) but it is challenging to realize practical large-scale neuromorphic architectures and read-out. Recently, two-terminal memristive devices have gained significant attention as the state of their internal resistance could indicate the history of the voltage across and/or current through the device (Yang et al., 2013). The memristive approach is promising in terms of low-energy, high-density memories and neuromorphic computing (Courtland, 2016), but as memristors are two terminal devices it may not be possible to simultaneously execute the signal transmission (computation or reading phase) and learning functions (writing phase). Metal NWs finds application mainly as interconnects and junction elements in crossbar memristors. Use of inorganic semiconductor NWs for HNN is an interesting direction.

Addressing the need for tactile HNN in e-skin, this paper presents a novel Neural Nanowire Field Effect Transistor (υ-NWFET) structure as the functional building block. The focus of the paper is on modeling, simulation and first validation with fabricated υ-NWFET structure prior to practical realization of a large area e-skin. This paper is organized as follows: The υ-NWFET device structure, working principle related to a biological neuron, and specific advantages of using NWs for HNN are presented in the Section υ-NWFET Based Neuro-Mimicking e-skin. Various modeling and simulation tools, and device fabrication methodology are presented in Section Methods. The results of modeling, simulation and fabrication are presented in Section Results along with experiment of e-skin integrated on a 3D printed robotic/prosthetic hand. Section Discussion discusses overall implementation and study of impact of fabrication related variability over HNN performance. The results are summarized in the concluding Section Conclusions.

## υ-NWFET based neuro-mimicking e-skin

**Figure 2:**
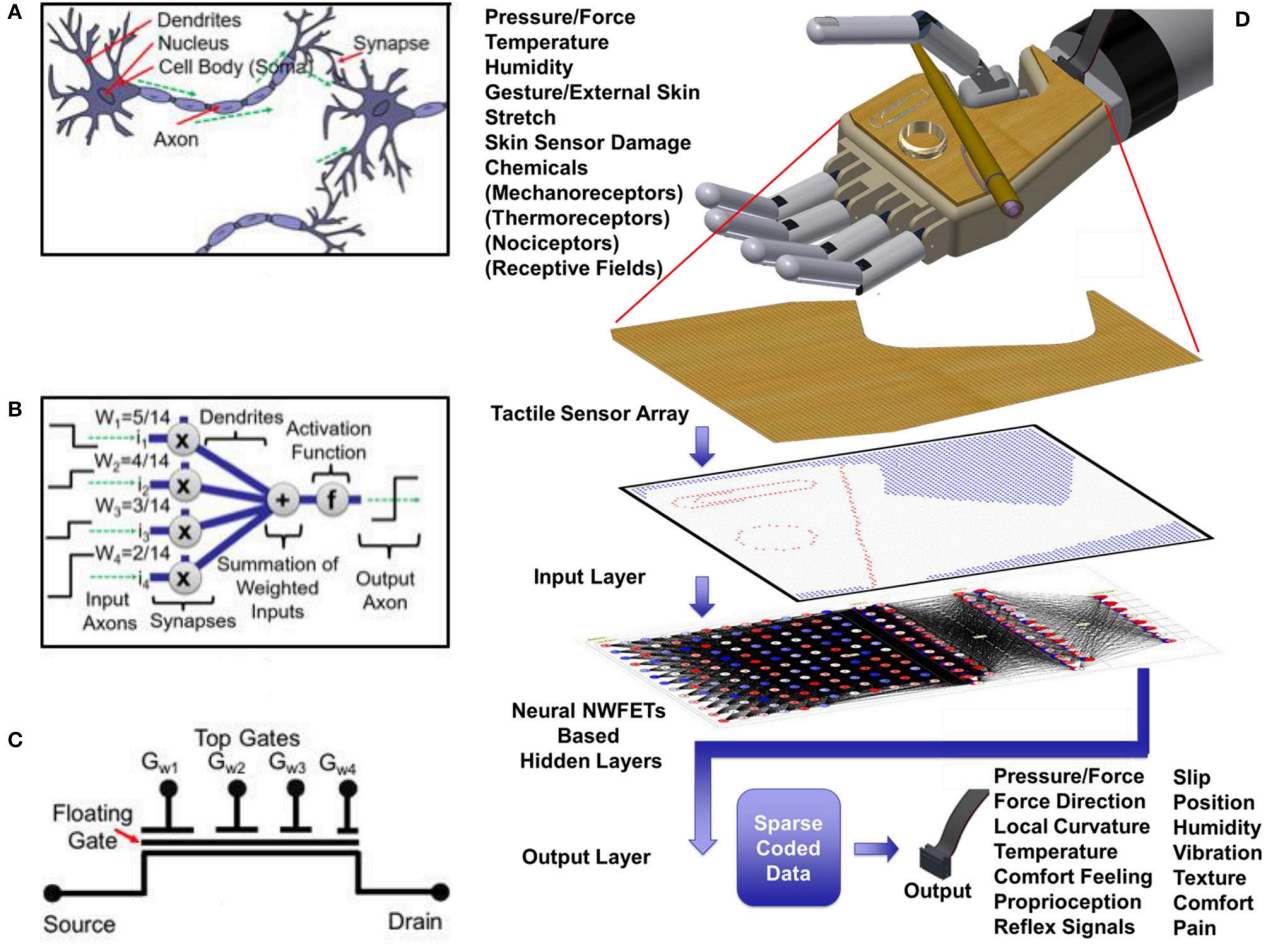
E-skin neural element and proposed tactile data processing scheme: **(A)** illustration of a biological neuron, **(B)** block diagram of an artificial neuron corresponding to the implemented weights. Complementary υ-NWFETs followed by an inverter are needed to realize this function, **(C)** symbolic representation of υ-NWFET, and **(D)** illustration of υ-NWFET based tactile e-skin covering an artificial hand. The simulated tactile signal is shown as stimuli to the input layer of υ-NWFET based network for coding of tactile information.

A simplified representation of biological and artificial neurons are shown in Figures 2A,B, respectively (McCulloch and Pitts, 1943; Goldstein and Brockmole, 2016). The υ-NWFET devices (symbol in Figure 2C and structure in **Figure 5A**) imitate the working of biological neuron in a simplified manner. The structure of υ-NWFET (**Figure 5A**) is a variant of a neuron MOSFET with NWs providing the functional channel region (Ishii et al., 1992; Shibata and Ohmi, 1992; Taube et al., 2016). The main floating gate, modulating the channel current is capacitively coupled to several gates. The overlap width of the individual gates over the floating gate determines the initial synaptic weight of the neural input on which further schemes of plasticity operates. This imitates the synaptic summation of weighted inputs in the cell body (soma) of the biological neuron or the artificial neuron. The activation function is performed at circuit level as discussed later in Section Circuit Modeling. It may be noted that the biological neural systems also have the plasticity or synaptic modulation, which reflects their ability to strengthen or weaken the synaptic weights over time. This modification of weights results in various forms of memory [namely, Sensory Memory, Short Term Memory (STM) and Long Term Memory (LTM), (Atkinson and Shiffrin, 1968)] at different hierarchical levels of the neural network. The sensory memory lasts for fraction of a second and is associated with local distributed computation involved in the tactile, smell or vision sensory system. Related to tactile perception, sensory memory plays a role in the local distributed computation such as force direction estimation, local curvature estimation, downstream reductions. Sensory memory on further hierarchical levels leads to STM which typically lasts for few seconds to a maximum of 30 s. These STMs gradually get transformed to LTMs at higher perceptual levels of neural network which can last up to lifetime. The υ-NWFETs based circuits presented here could exhibit similar behavior as discussed later in Section Circuit Modeling.

The working of υ-NWFET can be explained with modulation of the source-drain output curent by the voltage mode weighted summation of all input voltages to individual gates (*V*_*G*_*w*_*i*___). Voltage-mode summation through an insulating dielectric has significant advantage over current-mode summation as the standby power dissipation can ideally be avoided. The voltage in the floating gate (*V*_*FG*_) is given by:

(1) $$\begin{array}{r} V_{FG}=\frac{\sum_{i=0}^{3}C_{w_{i}}V_{G_{w_{i}}}}{C_{FG}+\sum_{i=0}^{3}C_{w_{i}}}+V_{Offset} \end{array}$$

Where *C*_*w*_*i*__ corresponds to the capacitance between the individual gate and the floating gate and determines the weighing factor *w*_*i*_. *V*_*G*_*w*_*i*___ corresponds to the voltage at each gate, *V*_*Offset*_ arises from any non-ideal charges such as fixed-oxide or interface trap charges (Shashank et al., 2011; Robinson et al., 2013).

The schematic illustration of proposed biomimetic tactile sensory neuro-system is given in Figure 2D. It consists of a prosthetic hand covered with a tactile e-skin; the simulated/measured tactile signal are read out by the receptive sensors to the input sensory layer of the υ-NWFET based neural network for sparse coding of tactile information. In bio-mimicking hardware, the sparse-coded output should comprise of encoded information such as pressure/force and temperature spatial and time distribution, force direction, local curvature, vibration, slip, humidity, comfort feeling, proprioception, reflex signals, pain, gestures. As a demonstration, in this paper, sparse coding of gesture direction has been presented based on υ-NWFET array architecture. Modeling was carried out to understand whether NW could be effective as a channel material for a neuron MOSFET as compared to the implementation with bulk material (Shibata and Ohmi, 1992). A combination of p-and n-channel υ-NWFET cascaded with a CMOS inverter has been demonstrated to represent a neuronal element. With multiple synaptic inputs and an output, the proposed structure will contribute toward building a computational architecture inspired by biological systems. An integrated hardware-realized neuromorphic tactile system could mimic or simulate biological activity and lead to unidirectional or bidirectional bio-electronic interfaces.

## Methods

**Figure 3:**
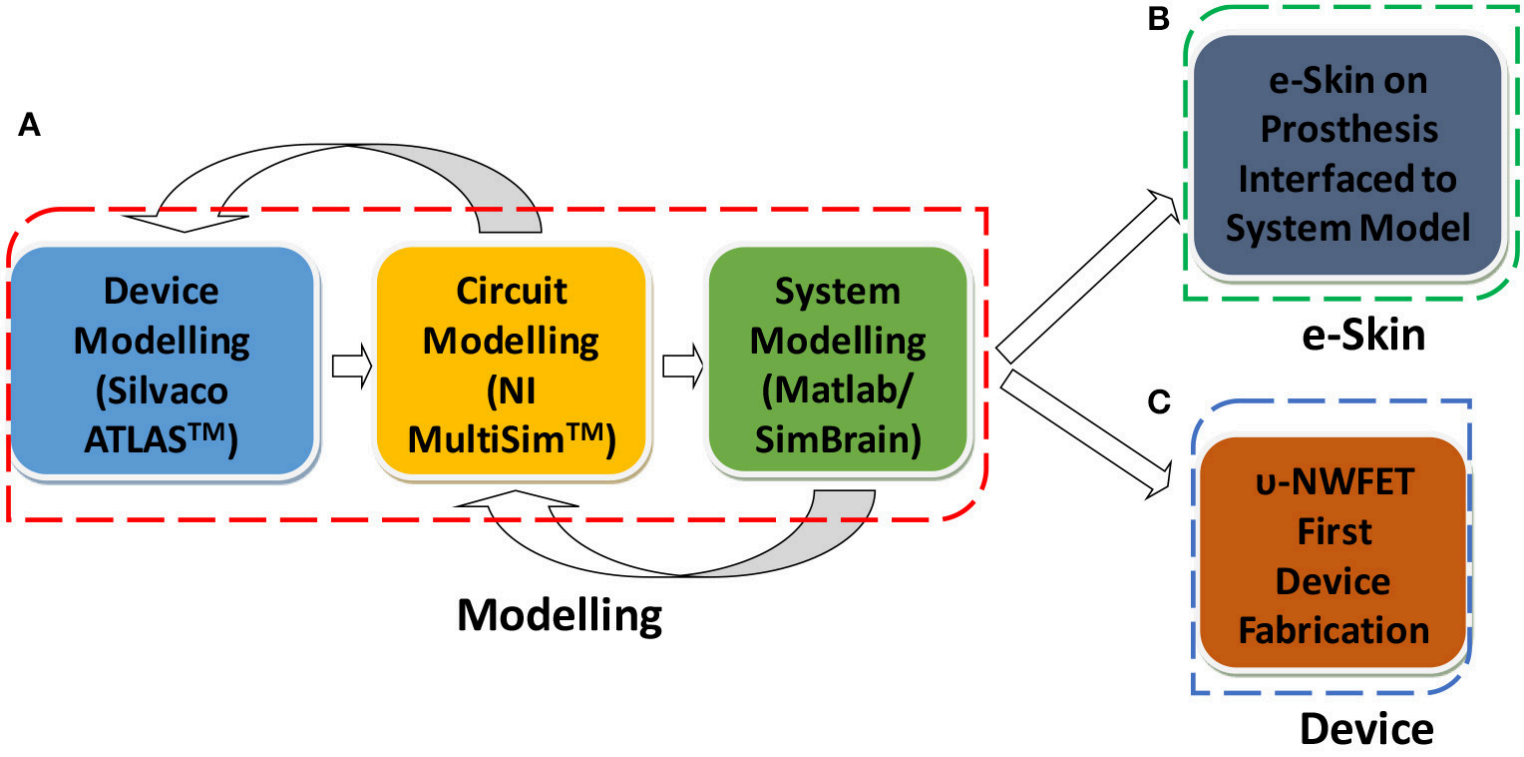
The work flow of the research presented in this paper. **(A)** Three levels of modeling. **(B)** e-Skin. **(C)** Device fabrication.

The viability of Si-NWs as an active material for HNN has been investigated through modeling and simulation, followed by the fabrication of first υ-NWFET device and tactile e-skin interface. The work flow of methodology used in this paper is summarized in Figure 3 and described below in detail.

### Modeling and simulation

**Figure 4:**
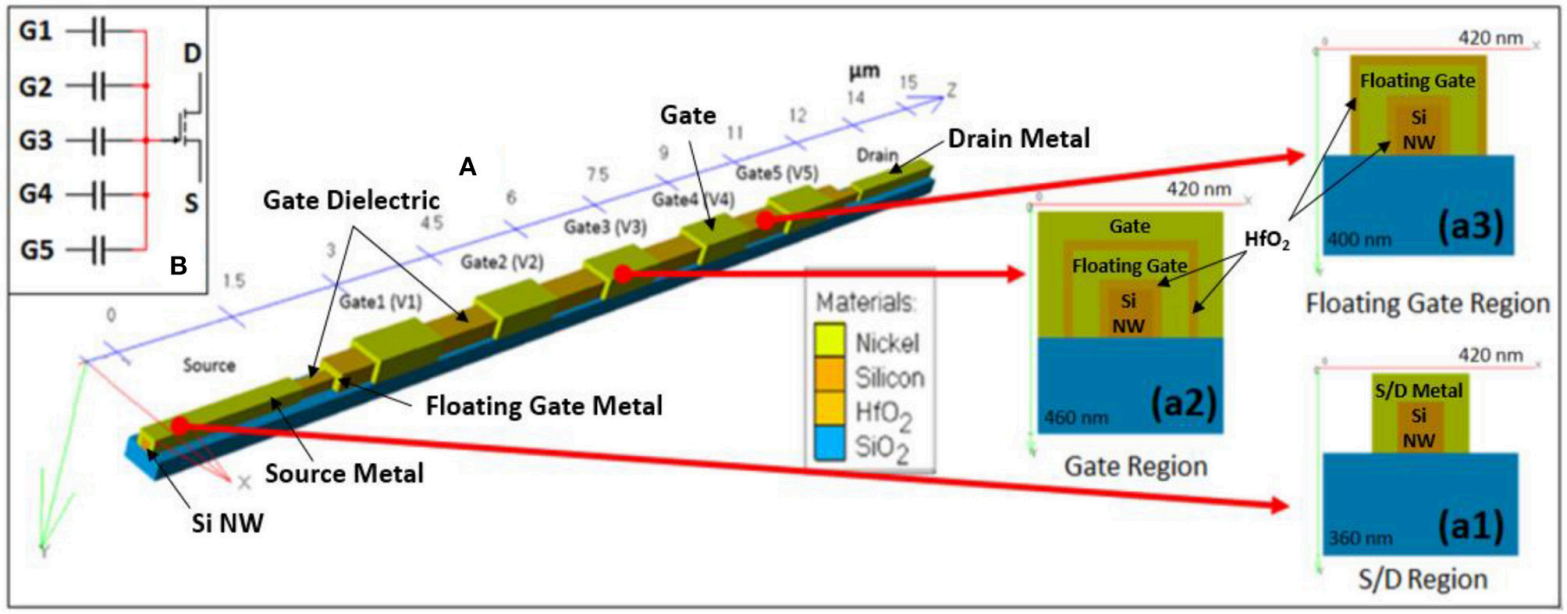
Modeling of υ-NWFET device. **(A)** 3D Structure of a simulated floating gate υ-NWFET. **(a1–a3)** Cross-sectional view of the υ-NWFET at drain/source region, floating gate and gate region. **(B)** Equivalent circuit model of a υ-NWFET.

The structure of proposed υ-NWFET device is shown in Figure 4. The modeling and simulation of the proposed approach was carried out at device, circuit and systems levels as shown in Figure 3A. The software tools used for this purpose (also shown in Figure 3A) include Silvaco ATLAS™ for device modeling, National Instruments (NI) Multisim™ for circuit modeling and Matlab and SimBrain for training and testing (offline and real-time) of the system model of NN. The implementation of υ-NWFET at device and circuit levels are illustrated in Figures 4A,B (top left inset) respectively. As shown in Figure 4A, the active channel region of the υ-NWFET consists of a p-type Si-NW with width, height and length (w × h × l) of 100, 100 nm and 15 μm, respectively. The channel region has p-type doping concentration of 10^14^ cm^−3^ and n-type source/drain doping concentration of 10^20^ cm^−3^. Ni was used for floating gate, top gate and source/drain contacts in this simulation. Of the total 15 μm length of the NW, the channel length corresponds to 10 μm. The simulated υ-NWFET comprises of five gates as input. The cross section of the simulated structure at the drain/source, gate and floating gate area are represented in Figures 4a1–a3, respectively. A 20.5 nm thick high-K dielectric such as HfO_2_ (or Al_2_O_3_), which corresponds to an effective oxide thickness (EOT) of 4 nm, was used between the input gates and floating gate and between the floating gate and the channel. The gates cover the NWs from three sides forming a tri-gated configuration. The individual gates' span is 1 μm with 1 μm separation gap between them. The υ-NWFET device simulation was carried out in ATLAS-3D to solve the fundamental semiconductor physics equations in three dimension. Further, the concentration dependent and the field dependent mobility models, and Shockley-Read-Hall (SRH) recombination model were defined to be solved by the solver. The default material parameters of Si were used in the solver (details given in Supplementary Section 2) while material parameters relevant to the simulation were given as input for user defined materials such as HfO_2_ (or Al_2_O_3_) and Ni. The dielectric permittivity for HfO_2_ and work function for Ni used in this work are 20 and 5.01 eV respectively. Fixed oxide charge density of 10^11^ cm^−2^ was defined between semiconductor/dielectric interface. Newton method was used to solve all the equations related to device simulation. All circuit simulations were carried out in Multisim™ with υ-NWFET device model implemented as a modified level-3 BSIM NW MOSFET model. This model is similar to the one used by Lee et al. (2009), except that the Schottky contact in source and drain were not considered as we have used heavily-doped source/drain junctions in this work. The parameters such as effective oxide thickness of high-K dielectric, electron and hole mobility, effective width and length etc., which were used in the device simulation, were also used in the circuit simulation (Refer to Supplementary Section 2).

### Fabrication and characterization of υ-NWFET device

**Figure 5:**
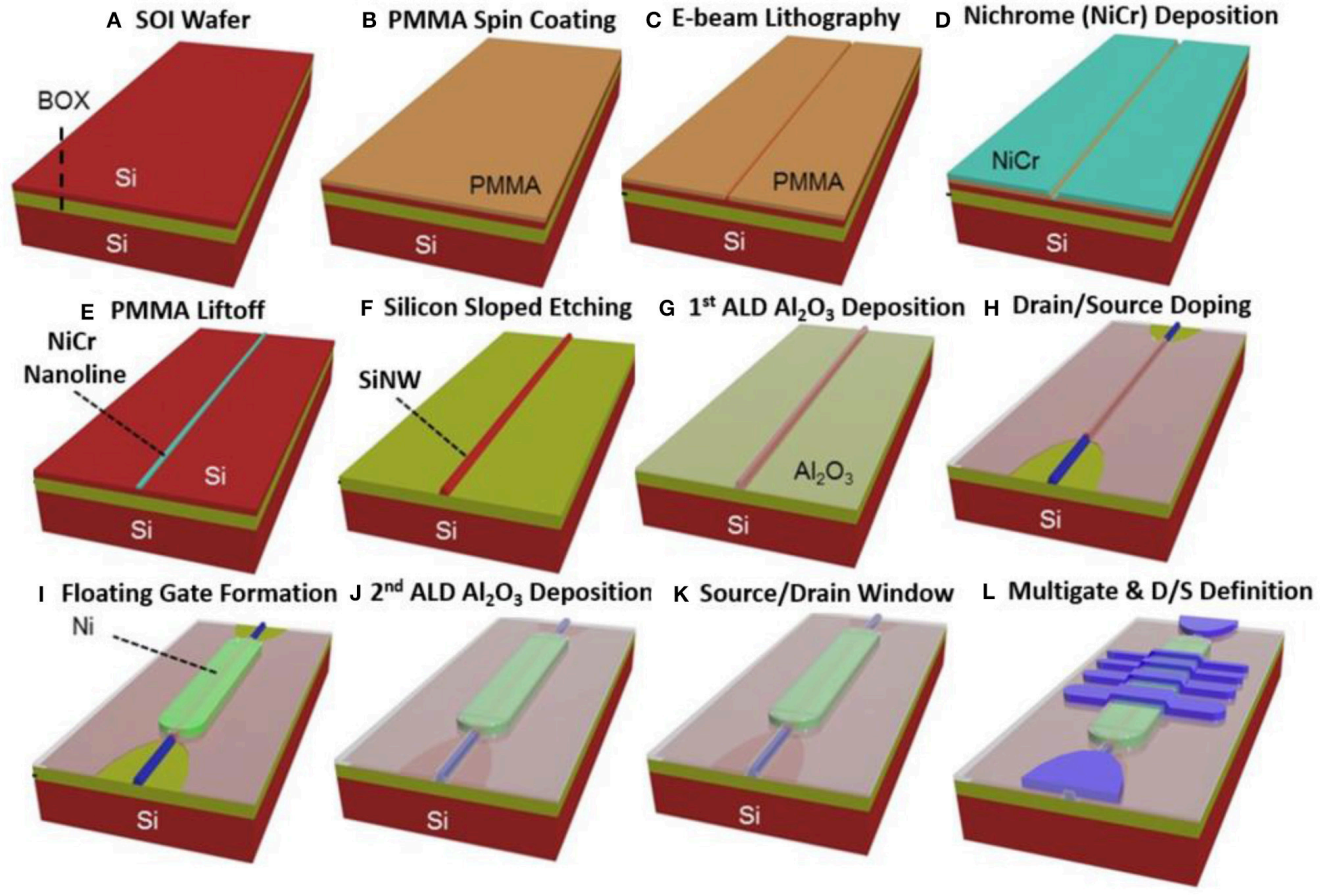
Schematic illustration of fabrication steps for υ-NWFET, using a SOI wafer as substrate **(A)**, and comprising **(B)** PMMA spin-coating, **(C)** e-beam lithographic patterning of lines on PMMA, **(D)** deposition of NiCr on the pre-patterned substrate, **(E)** PMMA lift-off, **(F)** Si sloped etching resulting in a trapezoidal shape Si NW, **(G)** ALD deposition of Al_2_O_3_ on top of the NW, **(H)** doping of drain and source, **(I)** Deposition of floating gate, **(J)** ALD Al_2_O_3_ deposition, **(K)** Definition of Source/Drain windows, and **(L)** Lift-off based patterning of Drain, Source and Multi-gates.

The fabrication steps carried out for realizing the υ-NWFET are shown in Figure 5. The device was realized on a Silicon-On-Insulator (SOI) wafer using standard top-down fabrication steps. The SOI wafer with the active layer thickness of 100 nm over buried oxide (BOX) of thickness 3 μm has been used as a starting material (Figure 5A). The supporting bulk Si had a thickness of ~626 μm. The active thin layer is p-type doped with boron has a resistivity of 14–22 Ω-cm. Here, electron beam lithography (EBL) has been used to define the pattern, with a double layer of PMMA2010 4% and PMMA2041 4% as the e-beam resist (Figure 5B). After EBL exposure (Figure 5C) and development, NiCr metal film of thickness 50 nm was deposited using an electron beam evaporation technique (Figure 5D), followed by a standard lift off process (Figure 5E).

The resulting NiCr nanoline (200 nm wide) acted as the hard mask during dry etching to get Si NWs. During dry etching process, a mixture of semiconductor grade SiCl_4_ and Ar gas was introduced in Reactive Ion Etching (RIE) system. The etching process used an optimized recipe to obtain a tilted slope of etch with nearly 45° angle. A mixture of 7.5 sccm SiCl_4_ and 15 sccm Ar was introduced in the etching process. This resulted in a trapezoidal shape which led to conformable coverage during subsequent processes. The hard mask was then removed by using NiCr etchant. A high-k dielectric layer (Al_2_O_3_) of thickness 50 nm was deposited conformably over the Si-NWs (Figure 5G) using atomic layer deposition (ALD) to insulate the forth-deposited Ni floating gate later in step shown in Figure 5I. This was followed by source drain doping and actuation. The source and drain were doped into targeted p+ doping concentration of ~10^20^/cm^3^. Since the starting substrate is p-type the above step resulted in a depletion mode υ-NWFET. Although this contrasts with the enhancement mode FET explained in the simulation section, it still serves the purpose when it comes to demonstrating the working of the υ-NWFET. The morphology of Si-NW was characterized by using AFM before and after Al_2_O_3_ deposition (Figure 5G) and doping (Figure 5H). Thereafter, a 30-nm thick Ni film was deposited over S1818 photoresist on an Al_2_O_3_ layer. This was followed by lift-off to obtain a floating gate for the neural FET (Figure 5I). The floating gate was encapsulated with another 30-nm thick Al_2_O_3_ layer deposited on top by ALD (Figure 5J). This was followed by the definition of source, drain and multi gate electrodes with 100 nm/10 nm Pt/Ti evaporated over UV lithography patterned photoresist and lift off (Figures 5K,L). Finally, the devices were sintered in forming gas (5% H_2_ + 95% N_2_) at 400°C for 20 min to conclude the device fabrication process. Since the capacitance plays a crucial role in the υ-NWFET, the Pt/Ti-Al_2_O_3_ (80 nm)-Si stack was studied using Capacitance-Voltage (C-V) characterization with a Keysight 1,520 A capacitance measure unit. The Current-Voltage (I-V) characteristics of υ-NWFET was also measured using Keysight 1,500 A semiconductor parameter analyzer. Analysis of the I-V characteristics indicates some device induced variation in gate weights, which could potentially lead to variations in the performance of the HNN.

### Fabrication of tactile sensitive e-skin

To demonstrate the real-time working of proposed approach, we fabricated a flexible and transparent e-skin comprising of 6 × 6 capacitive tactile sensor array and integrated over 3D printed hand developed in house. This is a step toward our future goal of obtaining a large scale υ-NWFET based printable electronic skin. The skin was interfaced with the SimBrain model through a capacitive array readout circuitry. The capacitive touch sensor array and the readout circuit was similar to our recently reported work (Núñez et al., 2017). However, in the present case we have used laser-ablation based patterning of indium tin oxide (ITO) on polyethylene terephthalate (PET) substrate instead of blade-cutting based patterning of Graphene on Poly-Vinyl Chloride (PVC) substrate. The touch sensor layer was fabricated using commercial ITO coated PET sheet from Sigma Aldrich, comprising of ITO film of thickness 130 nm and sheet resistance of 60 Ω/□ over PET of thickness 200 μm. The 6 × 6 touch sensor array has interlaced diamond patterns of ITO over an area of ~3 × 3 square inches. The sensing structure was designed to cover the palm of 3D printed prosthetic hand. Finally, the tactile sensitive e-skin was interfaced to SimBrain model and was tested in real time. To achieve this, the source code of SimBrain was modified to include a capacitive e-skin readout module programmed in Java. This final model was also used to understand the potential impact on neural function of the resulting network due to the deviation in gate weights arising from the line-edge roughness during fabrication, as described in previous subsection.

## Results

### Modeling results

#### Device modeling

**Figure 6:**
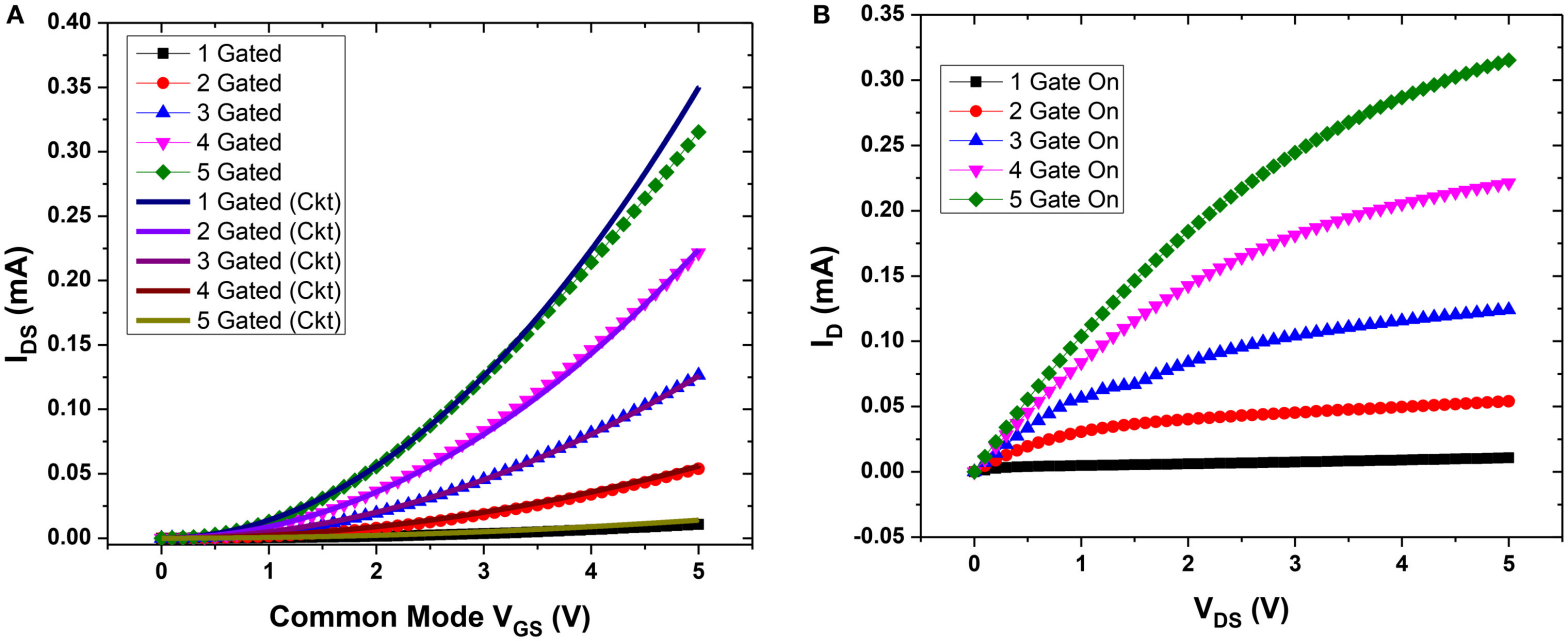
**(A)** Transfer characteristics from simulation of υ-NWFET device (dotted) and circuit (continuous) based on them. **(B)** Drain current (I_D_) vs. D-S voltage (V_DS_) as the gates are turned on one by one.

The weighted sum of voltages in the input gates determines the current *I*_*DS*_ between the drain and source. Figures 6A,B show the transfer and output characteristics of υ-NWFET respectively as the gates are turned-on one by one. In Figure 6A, the V_GS_ sweeps were carried out with common mode V_GS_ applied to a single gate and then progressively up to five gates. Working in the enhancement mode, the υ-NWFET is normally off. As the common-mode voltage is applied to two or more gates, the effective voltage in the floating gate of the transistor (Equation 1) increases and results in an increased current flow *I*_*DS*_ through the transistor. Figure 6B, shows V_DS_ vs. *I*_*DS*_ characteristics as 5 V is applied progressively from one gate to five gates.

#### Circuit modeling

**Figure 7:**
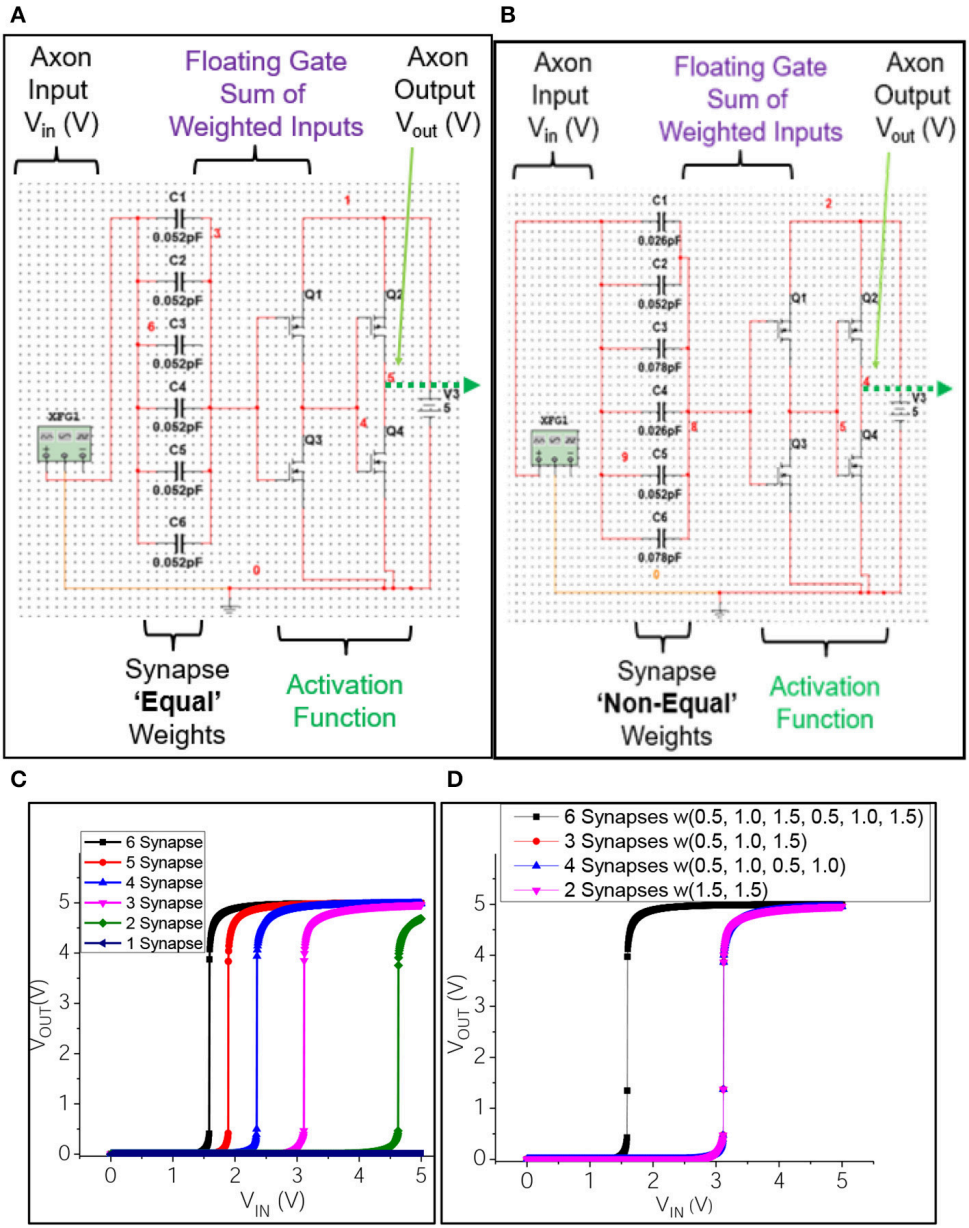
Equivalent circuit implementation of **(A)** equally and **(B)** non-equally weighted υ-NWFET based neurons and **(C,D)** their respective input-output characteristics.

The equivalent circuit of a υ-NWFET was also implemented in NI MultiSim with equal weights by connecting capacitances (corresponding to the dimensions of a υ-NWFET used in the device simulation) to an n-MOSFET (Figure 4B). To realize the logistic output typical of an artificial neuron, a complementary υ-NWFET-based inverter and complementary NWFET-based inverter were connected in series, as shown in Figure 7A to provide the activation function. All weights were kept equal, with a capacitance of 0.052 pF corresponding to a gate span of 10 μm. Rest of the parameters were unchanged from the simulated device in Figure 7. The output of this circuit (Figure 7A), shown in Figure 7C, shows that as the common mode input passes through more gates the neuron turns on at lower voltages. This indicates that the proposed υ-NWFET based neuron can provide a logistic output and could be used directly to realize neural network circuits—with the area of the capacitor's overlap with the channel determining the synaptic weight of the input. Figure 7B further highlights this with a circuit such as the one in Figure 7A, except that the non-equal gate weighted υ-NWFET neuron has been implemented. The common mode input is passed through synaptic weights (1.5, 1.5), (0.5, 1.0, 0.5, 1.0), (0.5, 1.0, 1.5), considering 3 as the maximum weight. The weights were realized with 3 capacitor values (0.026 pF, 0.052 pF, and 0.078 pF) at each of the n-type and p-type υ-NWFETs. Figure 7D shows that for all these cases (where weight is ~3), the neuron turns on at the same voltage of ~3.12 V. Also Figure 7D shows that the doubling of the total weight to ~6, results in the neuron turning on at ~1.59 V. Thus, a neural network implemented with a complementary υ-NWFET based inverter will have the weights hard-wired by the area of the gate span over the channel.

**Figure 8:**
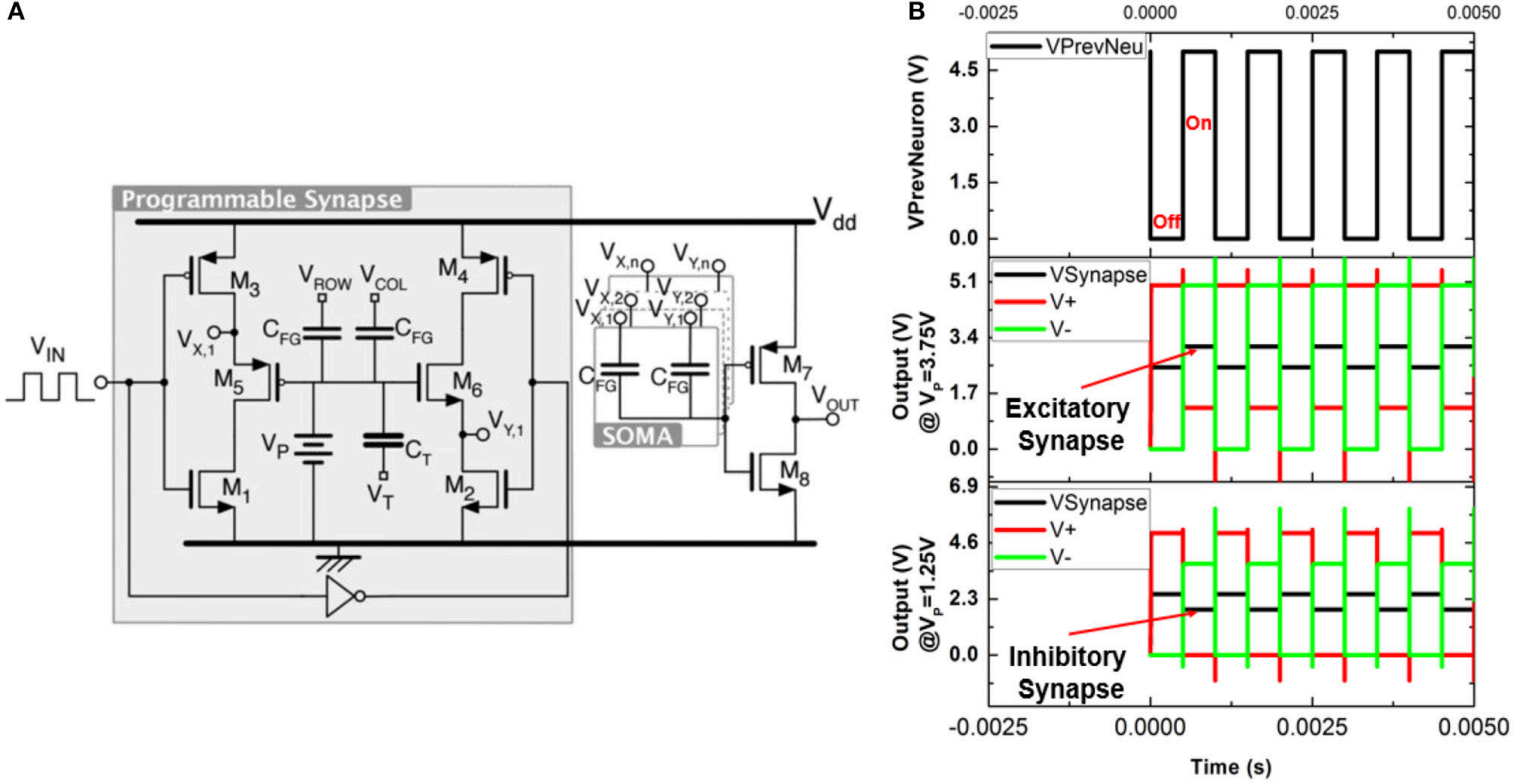
**(A)** Schematic and **(B)** output of a floating gate programmable synapse simulation.

**Figure 9:**
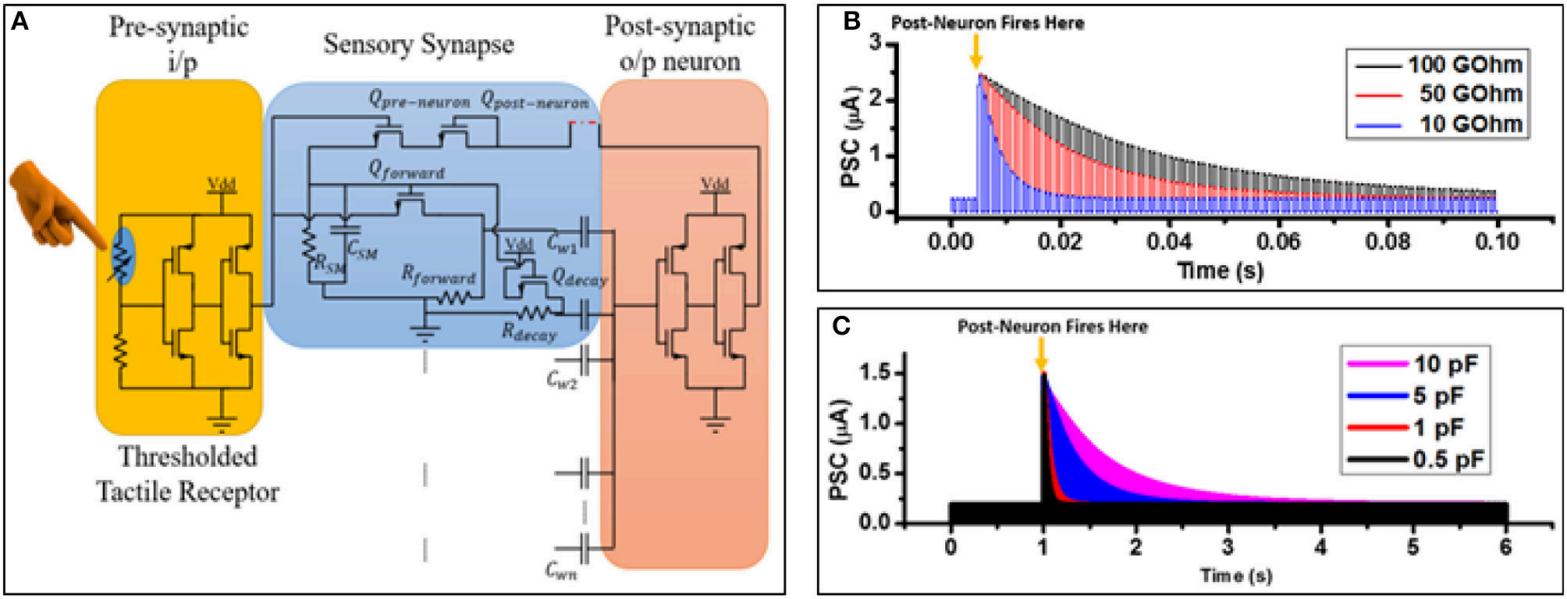
**(A)** Schematic of a neuron with sensory memory and adaptable toward STM and LTM. **(B)** Post synaptic current vs. time graph demonstrating sensory memory. **(C)** Post synaptic current vs. time graph demonstrating short term memory (STM).

For neuromorphic computing, plasticity or ability to modify the weights of the neurons is also needed. In this regard, the next two circuit level simulations (Figures 8, 9) are relevant as they indicate the steps toward synapses with plasticity using υ-NWFET approach. In both these cases, υ-NWFET based neurons act as a soma of the neuron while schema proposed for synapses are different. First approach (Figure 8) presents simulation of an EEPROM-like programmable υ-NWFET synapse to emulate the long term biological memory (Shibata and Ohmi, 1992; Yan et al., 2011). The second approach (Figure 9) shows simulations that are designed to emulate a sensory memory synapse utilizing passive components such as NW based resistors and capacitive structures along with υ-NWFET. Further directions have been proposed for this approach to extend it toward STM and LTM exploiting recent work on nanoionics transistors or memristors (Pillai and De Souza, 2017).

The schematic and the outputs of simulation of a neuron with a programmable synapse are shown in Figures 8A,B with complementary υ-NWFET and inverter to form a soma. The EEPROM-like programmable υ-NWFET forms an element of the synapse. Programming is carried out by selecting the V_ROW_ and V_COL_ and then applying the tunneling voltage V_T_ to let electrons tunnel through the tunnel capacitor C_T_ and program the floating gate voltage V_P_. This programmed voltage V_P_ determines the synapse weight and modulates the output V_Synapse_ (= (V_X,1_ + V_Y,1_)/2). It can be observed from Figure 8B that the gated p-MOS (left) and n-MOS (right) source followers are off whenever the output from the previous layer neuron (V_IN_) is off. This results in V_X,1_ (V+) = 5 V (i.e., V_DD_), V_Y,1_ (V−) = 0 V (i.e., V_SS_) and V_Synapse_ = 2.5 V (V_DD_/2), as shown in the output in Figure 8B. When the output from the previous layer neuron (V_IN_) is on, the V_Synapse_ results in an excitatory response for V_P_ > 2.5 V and an inhibitory response for V_P_ < 2.5 V. The graph in Figure 8B shows the result for V_P_ of values of 3.75 and 1.25 V corresponding to excitatory modulation and inhibitory modulation, respectively. This validates the υ-NWFET based circuit through simulation based on NI MultiSim. These neurons could be connected in various configurations to realize systems. However, one drawback of the above approach is that it requires 2 complementary υ-NWFETs per neuron, 2 complementary tunnel υ-NWFET and 4 NWFET per synapse. The EEPROM programming results in a non-volatile long term storage or long term memory. Further a high field is needed across SiO_2_ to achieve the tunneling, which could result in some reliability issues.

For tactile data processing, neurons with sensory memory like biological skin are preferred. In Figure 9A, we propose a circuit that takes advantage of NW processing to achieve neurons with sensory memory. The features include processing strategies to obtain a controlled array of NWs (McAlpine et al., 2005, 2007; Wang et al., 2008), the effectiveness of an array architecture (DeHon, 2003) and the inherent length of NWs. By taking advantage of the high aspect ratio of NWs, suitable resistance (R_SM_) and the capacitance (C_SM_) values can be obtained to realize a pattern designable RC delay operating in non-switched or switched mode. This could be further used to realize both sensory memory and short term memory. For example, a Si-NW with 100 Ω-cm resistivity, 100 μm length and square cross section with a width of 50 nm will have resistance ~40 GΩ. A monolithically integrated 20.5 nm thick HfO_2_-based dielectric between two 100 μm long and 500 nm wide metal NWs will give a capacitance of ~1.7 pF. Together these two components will lead to a RC delay of ~69 ms, which is of the order of a typical sensory memory (Atkinson and Shiffrin, 1968). Figure 9A shows the scheme of this approach with a sensory synapse implemented with a thresholder tactile sensory receptor (considered as a presynaptic input). The output of the presynaptic neuron passes through the voltage divider formed by the internal channel resistance of NWFET *Q*_*forward*_ and NW resistor *R*_*forward*_. This, along with the capacitance *C*_*w*1_, decides the synaptic weight. *C*_*w*1_ − *C*_*wn*_ might have equal values acting just as a summing unit (soma) as in Figure 7A or non-equal value as in Figure 7B. To avoid bootstrapping effect, it is best to have a depletion mode NWFET for *Q*_*forward*_. As per Hebbian learning, the correlation between firing of pre- and post-neuron strengthens the synaptic weight between these two neurons. This is achieved by the feedback path, from the output of post-neuron through *Q*_*post*−*neuron*_ and *Q*_*pre*−*neuron*_ to sensory memory element *C*_*SM*_. When the pre- and the post-neuron fire together, the latter charges the *C*_*SM*_ and causes the *Q*_*forward*_ NWFET's internal resistance to decrease. This eventually increases the synaptic weight between the pre- and post-neuron. Figure 9B shows a typical output when the sensory memory NW resistor's (R_SM_) value is changed between 100, 50 and 10 GΩ, for a pulsed pre-synaptic firing of a neuron with a duration of 500 μs. As soon as the post neuron fires (500 μs) along with pre-neuron, the *C*_*SM*_ gets charged. The excitatory post synaptic current decays exponentially as the *C*_*SM*_ discharges through R_SM_. The neuron will continue to get pre-synaptic input through the voltage divider between *Q*_*decay*_ and *R*_*decay*_. The actual effective capacitance contributing to the sensory memory is a combination of gate capacitance of *Q*_*forward*_ as well as C_SM_. Depending on the timing requirement of the sensory memory, C_SM_ could even be avoided, thus making use of the internal capacitance of *Q*_*forward*_. The output of the neuron (*V*_υ−*out*_) is a function of *V*_*FG*_, which depends on the gate inputs (*V*_*Gn*_) incident on the various capacitors as given by Equation (1). *V*_*Gn*_ is given by the internal resistance *R*_*Q*−*forward*_ of *Q*_*Forward*_ and the resistance *R*_*Forward*_:

(2) $$\begin{array}{r} V_{Gn}\left(t\right)=\;V_{\upsilon -in}\frac{R_{Q-forward}\left(V_{MEM}\left(t\right)\right)}{R_{Forward}}\left(1-e^{-\frac{t}{\tau 1}}\right) \end{array}$$

*R*_*Q*−*forward*_ depends on *V*_*MEM*_(*t*) across *C*_*SM*_ which is given by:

(3) $$\begin{array}{r} V_{MEM}\left(t\right)=\;V_{\upsilon -in}\frac{R_{SM}}{R_{TotReverse}}\left(1-e^{-\frac{t}{\tau 2}}\right) \end{array}$$

Where,

(4) $$\begin{array}{r} R_{TotReverse}=R_{Q-Pre-neuron}\;+\;R_{Q-Post-neuron}+R_{SM} \end{array}$$

To demonstrate the effectiveness of the proposed approach and to advance it to system level, a tactile information processing problem was simulated as explained in the next section.

#### System modeling

**Figure 10:**
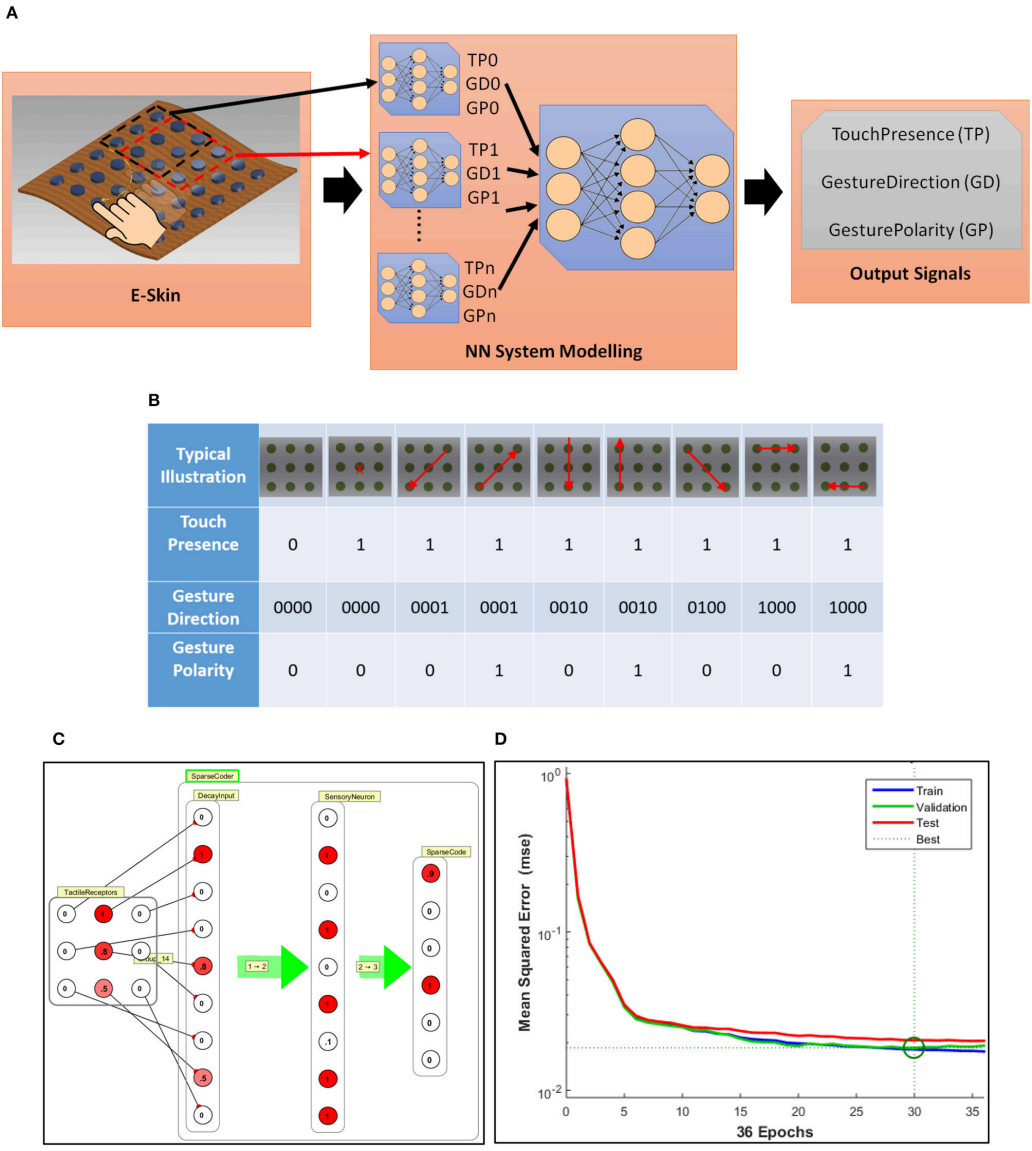
**(A)** Schematic illustration of sparse coding of tactile data from e-skin neural information. **(B)** Goal of the sparse coding. **(C)** Typical implementation of one Level 1 sub neural network cell on SimBrain; the output of the decay sensory neuron is passed to the input of the sparse coder which gives the sparse coded output. **(D)** Mean squared error vs. epochs during training of the network.

The schema of the sparse coding system shown in Figure 10A comprises of an array of tactile sensors (6 × 6) which acts as an input to the NN system model. The target of the current sparse coder is to encode the tactile input or gesture into three outputs “TouchPresence,” “GestureDirection,” and “GesturePolarity.” The outputs are considered as bits depending on whether the neuron is on or off. “TouchPresence” is a single bit output which signifies downstream reduction i.e., whenever one of the tactile sensor (out of the 6 × 6 sensors) is touched, the output should be on, as shown in the second column of Figure 10B. This could be used as an event driven triggering stage for triggering the higher stages of a neural network. “GestureDirection” is a 4-bit data output for which the values 0001, 0010, 0100, 1000 correspond to the directions NE to SW, N to S, NW to SE, and W to E respectively. The reverse direction for each case has the same output value for “GestureDirection” except that “GesturePolarity” is set to 1 instead of 0, as shown in Figure 10B. The model was implemented in two levels. The lower NN level (Simbrain snapshot shown in Figure 10C) acquires input from a 3 × 3 sub-array of the e-skin to a 9 thresholded tactile receptor decay neurons. Since, the feeling of gestures on the skin depends on sensory memory, the above application serves as an effective way of testing the proposed approach. Hence, the sparse coder was modeled as a combination of a Decay Neuron Network forming an input layer followed by a feedforward neural network. The simulations were performed on SimBrain (Tosi and Yoshimi, 2016). The decay time depends on the time of the sensory or short term memory decay in Equation (3). (Supplementary Section 3 shows the snapshot of the time series plot of decay of the simbrain simulation of four neurons with different decay constants). The decay neuron forms the input to a sparse coder. From a system viewpoint, this 3 × 3 sparse coder could be considered as a low-level cell. In a hardware implementation, this could be realized with NWs-based lower level cellular structure in the backplane of 3 × 3 tactile sensors sub-array. This approach enables achieving hierarchical upstream reductions. The level 1 sparse coder was modeled and trained in Matlab using the Levenberg-Marquardt method with 9 logistic hidden layer neurons corresponding to 9 sensory neurons feed forwarded to 6 output neurons. The input and target for training, validation and testing the sparse coder as per Figure 10B was generated using a Matlab code generating various tactile gestures based on a random number generator. A total of 5,000 samples were generated out of which 3,500 samples were used for training, 750 samples for validation and further 750 samples for testing the system. After training, the weight and bias matrices were transferred to SimBrain for testing and visualization. Based on the simulation, training and validation, it was found that the dataset was linearly separable and was implementable with a single layer neural network with the output converged to a mean squared error of 0.02 for linear output neurons-implying a zero error as with the logistic or binary output stage. Figure 10C shows the typical implementation on SimBrain where the output of the receptor is passed to the sensory neuron through the decay inputs. The sensory neuron and the higher level logistic neurons together perform the sparse coding of the input to outputs as in Figure 10B. The first 3 blocks in Figure 10C together mimic the functionality of the sensory neuron of the circuit given in Figures 9A, 10D shows the mean squared error vs. epochs during training of the network. The network converged within 30 epochs, giving a mean squared error of 0.02. In the next section, the interface of a tactile skin to a higher level NN (level 2) through many such level 1 cells to emulate tactile gesture recognition is presented.

### Experimental results

#### e-skin on prosthetic hand interfaced to system model

**Figure 11:**
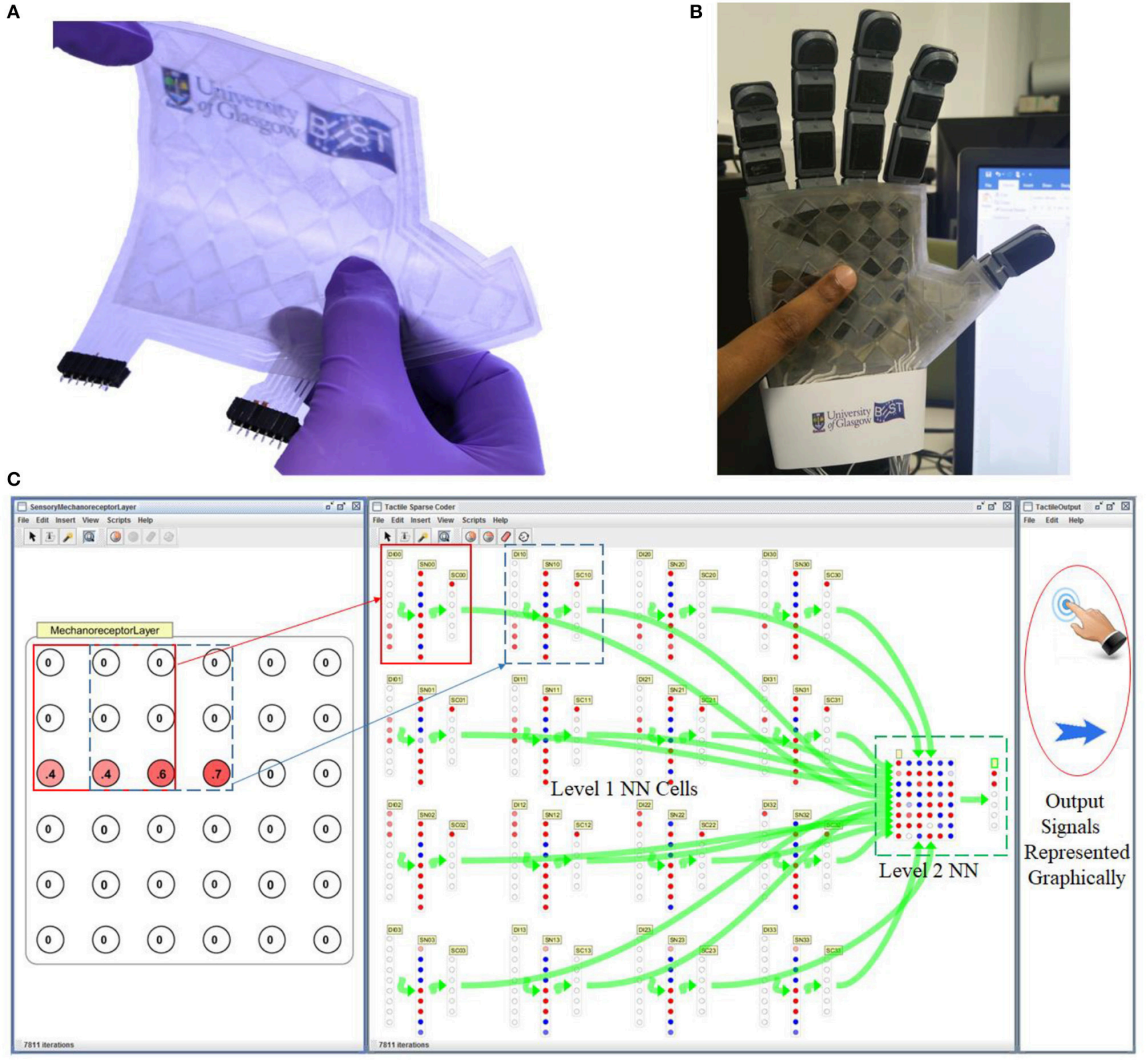
System model interface with e-skin on prosthetic hand **(A)** Flexible and transparent touch sensitive e-skin **(B)** Image of the prosthetic/robotic hand with e-skin **(C)** Snapshot of SimBrain model showing mechanoreceptors layer and associated sensory processing level 1 NN cells are connected to higher hierarchical level 2 NN block. Video of the demonstration in Supplementary Video 1.

The fabricated flexible and transparent touch sensitive e-skin is shown in Figure 11A. The fabricated passive tactile sensitive e-skin integrated on a 3D printed prosthetic/robotic hand (Figure 11B) was interfaced to the system model and tested in real time. Figure 11C shows the snapshot of SimBrain model showing mechanoreceptor layer comprising of 6 × 6 elements. The 3 × 3 overlapping window of receptor elements are connected to individual local processing level 1 NN cells as shown in Figures 10A, 11C. The output of all level 1 NN cells correspond to an array of 96 elements form the input layer for another hierarchical level of the feed forward neural network comprising of 48 neurons in the hidden layer and 6 neurons in the output layer. The value of the output layer of NN shown in Figure 11C indicates touch or direction events given in Figure 10B. The output signals corresponding to the value of the output layer are graphically represented in the third window of Figure 11C. The video of the demonstration is included in Supplementary Video 1.

#### Characterization of υ-NWFET

**Figure 12:**
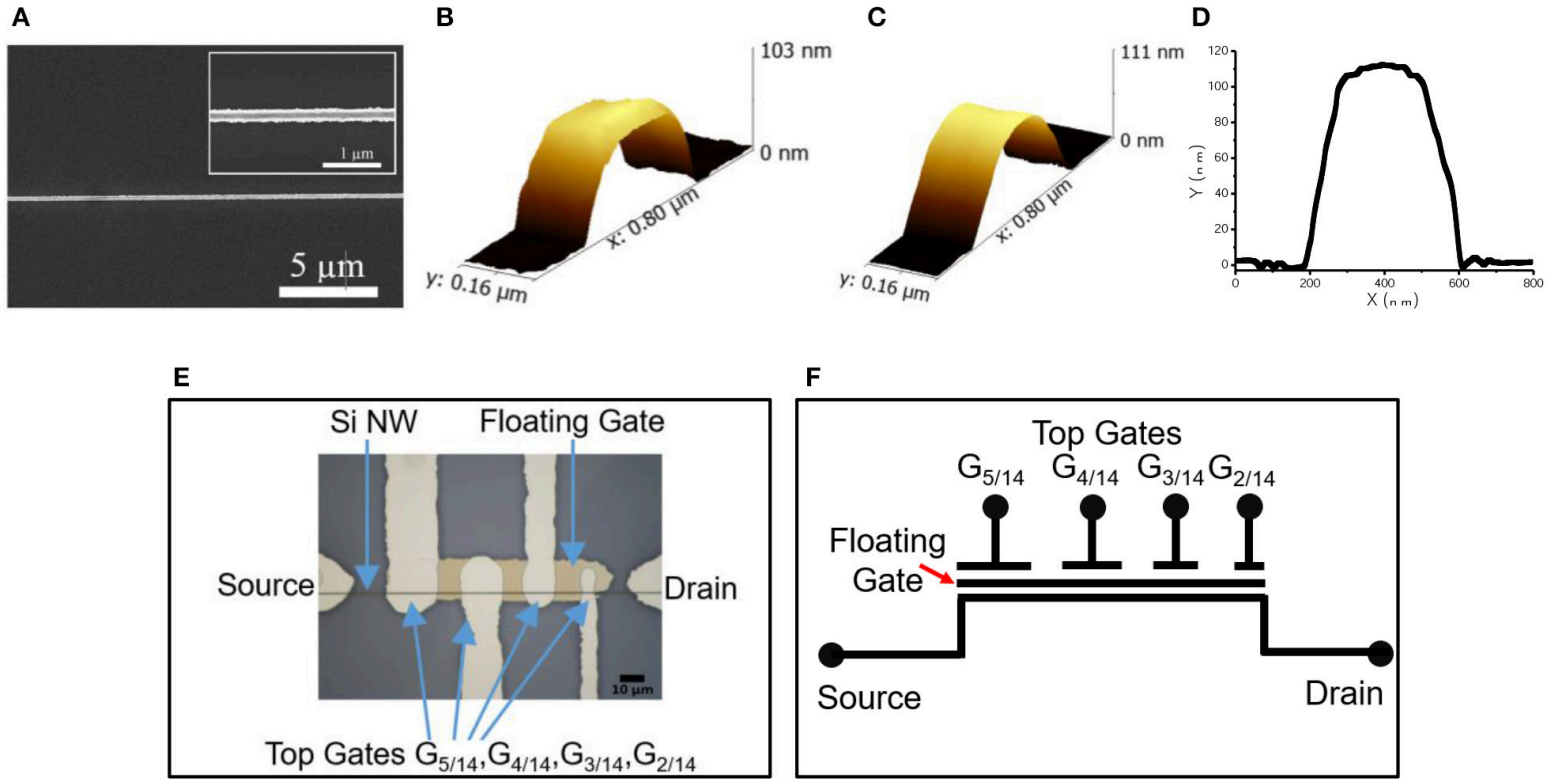
**(A)** SEM image of NiCr nanoline in low magnification. This pattern is subsequently etched into trapezoidal NWs (Inset: High magnification). Three-dimensional AFM scan of SiNW taken **(B)** before atomic layer deposition (ALD) and **(C)** after ALD & thermal annealing processes; **(D)** Line profile corresponding to the AFM image **(B)** where a ~45° trapezoidal structure is observed. **(E)** Optical microscopy image of the fabricated υ-NWFET. **(F)** Symbolic representation and weights of the fabricated υ-NWFET.

The structural and electrical characteristics of Si υ-NWFET are presented in this section. As shown in the SEM images in Figure 12A, the fabrication process (described in Figure 5A–l) results in a NiCr nanoline of width 200 nm which was used as a hard mask during dry etching for obtaining the Si NWs. After fabrication step shown in Figure 5F, the Si-NW was characterized by using AFM before and after Al_2_O_3_ deposition and doping (Figure 12B–D). The Si-NW has a thickness of ~100 nm after etching, as shown in the AFM image in Figure 12B. After ALD processing and doping, the surface is smoothened as depicted in Figure 12C. The optical microscopy image of a fabricated prototypical υ-NWFET is shown in Figure 12E. In the fabricated four-gated υ-NWFET, the gates spanned 25, 20, 15, and 10 μm over the floating gate electrode, result in synaptic weights of around 5/14, 4/14, 3/14, and 2/14 respectively as illustrated in Figure 12F. Here, C_FG_ is not included as the capacitance between the floating gate and the NW is negligible compared to the capacitances formed by the metal gates. Hereafter, the gates are referred, based on their synaptic weights, as G_5/14_, G_4/14_, G_3/14_, and G_2/14_.

**Figure 13:**
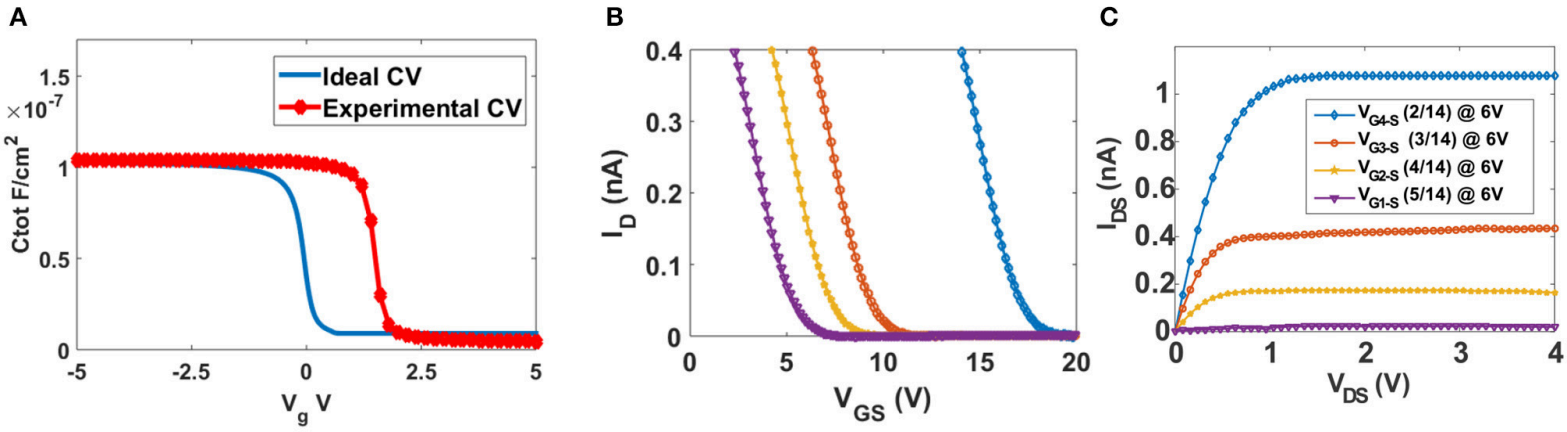
Electrical characteristics of Si υ-NWFET. **(A)** Ideal (blue) and experimental (red) C-V_g_ relationship. **(B)** V_GS_ vs. I_DS_ characteristics for G_5/14_ to G_2/14_ at V_DS_ = 4 V **(C)** V_DS_ vs. I_DS_ characteristics while applying 6 V to each gate, one by one.

The turn-off voltage of the υ-NWFET is influenced by the presence of various charges and interface trap density in the dielectric. The Pt/Ti-Al_2_O_3_ (80 nm)-Si stack was studied using Capacitance-Voltage (C-V) characterization with a Keysight 1520A Capacitance Measure Unit. The Capacitance was measured for a gate voltage in the range of −5 to 5 V at 1 MHz frequency with a 50 mV-rms a-c signal. Both the ideal C-V and the experimental C-V curves are plotted in Figure 13A. Here, we used a Matlab code to obtain the ideal C-V curve by solving Poisson's equation (Sze and Ng, 2006). The work function of the electrode (Ti), average doping concentration and the oxide thickness are defined as input parameters in this code. The value of flat band capacitance (*C*_*FB*_) was obtained from the ideal C-V curve at *V*_*g*_ = 0 V. This *C*_*FB*_ was used to get the flat band voltage (*V*_*FB*_) the experimental C-V curve (*V*_*FB*_ = 1.6 V). The fixed oxide charge density was calculated (*Q*_*OX*_ = −1.43 × 10^12^ e-cm^−2^) by finding the flat band voltage shift. The interface trap density *D*_*IT*_, calculated using the Terman method, was found to be in a range of 1.39–7.89 × 10^12^ eV^−1^cm^−2^. The ideal and practical threshold voltages (*V*_*th*_) are ~0.6 and ~2.2 V, respectively. In the case of a floating gate structure, the effective voltage needed on the floating gate (50 nm from channel) is less than the inversion voltage observed from the C-V characteristics. Further, the additional charges in the floating gate dielectric interface will result in deviation from the ideal turn off voltage (expected ~1.4 V). Figure 13B shows the V_GS_ vs. I_DS_ characteristics with voltage sweep applied to the gates of the υ-NWFET one at a time, while others kept at 0 V. Since the presented υ-NWFET works in depletion mode like a gated resistor, the channel depletes with an increase in the gate voltage, finally inverted, resulting in a decrease in the current. The dependence of the observed turn-off voltage (i.e., 6.1, 7.7, 9.8, and 16.8 V (rounded to 1 decimal point for gates 1–4 respectively) on the synaptic weight of each gates demonstrates the working of the υ-NWFET. Figure 13C shows the transfer characteristics of the υ-NWFET as each gate is given 6 V, one at a time. The gate with higher synaptic weight suppresses the current more compared to the gate with the lower synaptic weight in the order G_2/14_ to G_5/14_ with current reduced ~54 times from 1.0772 ± 0.01 nA to 19.6 ± 0.1 pA at V_DS_ = 4 V and V_GS_ = 6 V. The difference between the trend in the simulation presented in Figures 6A,B and actual data Figures 13B,C could be attributed to the fact that the υ-NWFET presented here works as a gated resistor in depletion mode in contrast to the simulation. The gates with non-equal width were given voltage one at a time while the rest were at zero potential in contrast to the simulated device. Further, from the current values, the contacts appear to be Schottky-type, whereas simulation considers a perfect ohmic contact. The early saturation observed in this long channel υ-NWFET could also be attributed to the saturation in one of the Schottky mode contacts. Higher performance could be obtained in a sub-50 nm υ-NWFET with optimized contacts. The results herein clearly indicate the expected neuronal function from the υ-NWFET device.

#### Effect of fabrication induced gate weight variability on the performance of system model

**Figure 14:**
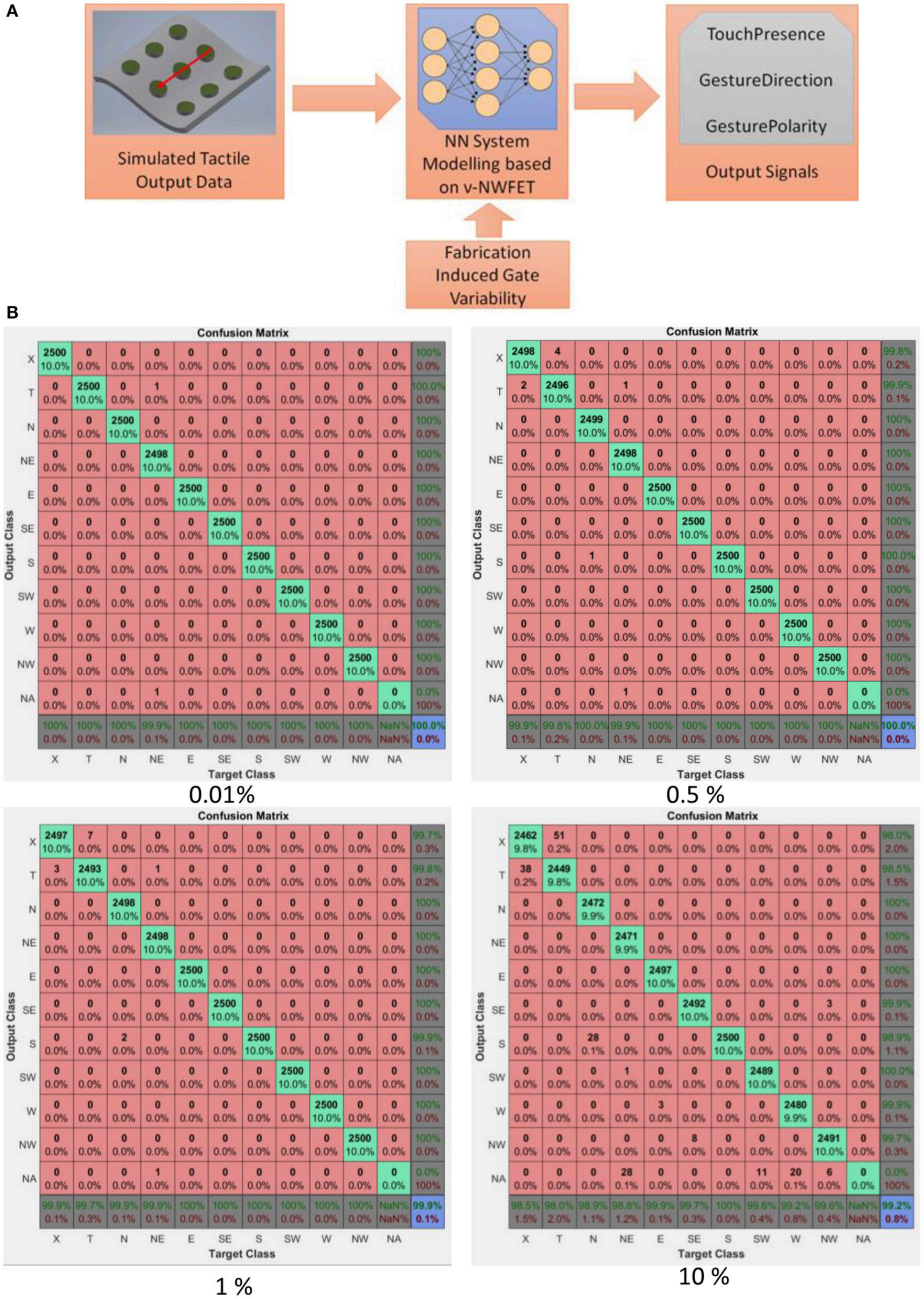
**(A)** Schema to study impact on neural function of the NN due to the deviation in weights. **(B)** Effect of weight deviations on the NN's performance.

The system model given in Section System Modeling were used to understand the potential impact on neural function of the resulting network due to the deviation in gate weights arising from the line-edge ughness during fabrication (seen in Figure 12E). This is schematically illustrated in the Figure 14A. The line-edge roughness results in variation in the capacitances compared to the design value. By fitting the cut-off voltage obtained in the previous section, the experimental capacitances were obtained. The results are compared with the design capacitances in Table 1. A deviation of ~ <0.1% are observed between design and experimental capacitances. The weights of the system model were changed using a random number generator to maximum of 10% to check its effect on the sparse coder. The results are plotted as confusion matrices in Figure 14B. The class values in the x and y-axis namely, X, T, N, NE, E, SE, S, SW, W, NW, and NA corresponds to No Touch, Touch, North, North East, East, South East, South, South West, West, North West and Not Applicable respectively. With 0.01% weight deviation, only 2 out of 25,000 classifications were misclassified. For 0.5, 1, and 10% deviations the number of samples that were misclassified were 9, 14, and 2,546 samples out of 25,000 were misclassified which shows the inherent robustness in NN.

**Table 1:** Comparison of designed capacitances with experimental capacitance for various gates.

**Capacitance**	**G_5/14_**	**G_4/14_**	**G_3/14_**	**G_2/14_**
Design	1.3812pF	1.1050pF	0.8287pF	0.5525pF
Experimental (Fit)	1.3932pF	1.1037pF	0.8672pF	0.5059pF
Deviation (%)	0.00869	0.00118	0.04887	0.08434

## Discussion

The υ-NWFETs based approach for realizing HNN has several advantages for tactile data processing in electronic skin (e-skin). For example, it allows implementation of neural circuits in a compact array architecture (DeHon, 2003). With good subthreshold control of a tri-gated or gate all around NWFETs (Kuhn, 2012), it would also be possible to develop highly power efficient devices or circuits. Further, the possibility of printing NWs (Shakthivel et al., 2015; Yogeswaran et al., 2015; Navaraj et al., 2017) means with υ-NWFETs it will be possible to develop bendable or conformable systems, which is much needed for better integration of e-skin on curved surfaces such as the body of a robot or prosthetic hand (Dahiya et al., 2015). Such e-skin could have printed υ-NWFETs in the backplane (Yogeswaran et al., 2015; Shakthivel et al., 2016) to communicate with higher perceptual levels. It is possible to have 3D integration or stacking of NWs based circuit (Javey et al., 2007) and if such work is extended for e-skin then we may see more advantages, particularly, in terms of mimicking biological tissues and brain. The integration of υ-NWFET based neural processing circuits with NW-based neural recording/mapping and stimulation circuits is another direction that could significantly advance the research in neuro-prosthetics, bio-neuro interfaces and electroceuticals (Patolsky et al., 2006; Qing et al., 2010; Robinson et al., 2013; Thomson et al., 2017).

While the direction is interesting and promising, there are significant challenges associated before realizing a fully biomimicking artificial tactile skin. The tactile data processing in biological e-skin is much complicated and has complex pathways. The notion of neurons being represented as entities performing weighted summation followed by actuation itself is a significant approximation far from a real neuron, and a slightly more closer approximation uses time-domain differential equations to explain biological neuron's membrane dynamics and interaction (Marder and Taylor, 2011).

Biological neurons are highly energy efficient compared to most artificial implementation of neurons (Boahen, 2017). To achieve energy efficiency and better performance, a υ-NWFET should have lower leakage current, higher drive current and higher on-to-off ratio. High-K dielectric used as a gate dielectric to avoid gate leakage while still having lower EOT ensures better coupling and control of gates over the channel. Further, the gates formed in a trigate configuration around the NW offers better subthreshold performance. On-to-off ratio of up to 2.6 × 10^4^ was achieved for the simulated device structure with all gates ON vs. all gates OFF. For packing more neurons per unit area and improving performance further, the υ-NWFET must be scaled in all dimensions such as the width, length of the NW, the gate span of each gates.

When such intense scaling is carried out, the process variations such as variations in doping concentration, NW dimension, gate width, line edge roughness may influence the synaptic weights and performance of the neural network. However, neural networks are known to be inherently fault tolerant and robust. In the presented work, comparison of designed capacitances with experimental capacitance shows a maximum fabrication induced weight variation ~0.1%. Allowing up to 1% change in the weights of the system model lead to misclassification of the data set by only ~0.06% which shows the robustness of NN for such applications.

The various circuit approaches presented could be used as sub-components for neuro-mimicking tactile e-skin and based on system requirement, choice can be made between hardwired-neuron with no plasticity (as in Figure 7), neuron with plasticity having sensory memory or STM (as in Figure 9) or LTM (as in Figure 8) to be used at different hierarchical levels of the tactile sensing NN. Synaptic plasticity finds application both in data storage/memory as well as neural computation. In the first approach, i.e., hardwired-neurons, learning and circuit/layout synthesis will be through software tools which will be followed by practical fabrication forming a hardwired neural network. By introducing additional plastic synapse schemes (as in Figures 8, 9), the weight could be modulated over the initial value set by the capacitances. In this case the synaptic weight initially set by the capacitances could be considered as a phyletic memory (Fuster, 1997) because it is hardwired over which further schemes of plasticity operates. Such an approach could be considered as a semi-plastic neural network. For such a network, the quantization arising from layout synthesis (For example rounding-off the weight equivalent capacitance dimensions to 1 μm) followed by lithography process for fabricating the capacitors will lead to k-levels of possible discrete synaptic weights (Obradovic and Parberry, 1992; Obradović and Parberry, 1994). The approach proposed in Figure 9 results in a programmable synapse, which could be used to implement hardware-in-the-loop learning. It is to be noted that in the initial stage of tactile sensing the sensory data need to be stored only for a short time and hence a neural circuit with sensory memory is sufficient for earlier tactile data handling as in Figure 9. This circuit could be further modified for higher hierarchical levels to have STM and LTM associated plasticity. A transitionary circuit from sensory memory to STM can be achieved either by replacing the *C*_*SM*_ with an element of higher value (as in Figure 9C) or by replacing *Q*_*forward*_ with a nanoionic-like transistor (Pillai and De Souza, 2017) for use in higher hierarchical level of neural network beyond tactile skin. Beyond that, increasing *C*_*SM*_ may not be a practical option as it becomes bulkier in the process of realizing longer times. Possible strategies for transition from STM to LTM at different stages of the network include replacing *Q*_*forward*_ by a nanoionic-like transistor (Pillai and De Souza, 2017), or with a NW-based programmable floating gate transistor (Yan et al., 2011), or replacing *R*_*forward*_ with a memristive device (Yang et al., 2013).

The system model interfaced with a flexible and transparent touch sensitive e-skin with 6 × 6 tactile elements and tested in real-time demonstrates the working of the proposed approach. However, since the SimBrain model involves software NN programmed in java to mimic the HNN, a delay of ~1.12–1.54 s was observed per cycle for the implementation with Intel® Core™ i7-4500U CPU @ 2.4 GHz with 8GB RAM and a delay per cycle of ~0.394–0.470 s was observed for implementation in Intel® Core™ i7-4790K CPU @ 4 GHz with 32GB RAM. While this could be improved by approaches such as use of GPU, dedicated HNN such as the proposed approach will be optimal for real-time tactile data processing. The delay with software NN will be much substantial if the number of tactile elements are increased for example to a human palm ~18,675 MRs as shown in Figure 1. Further advancements in the system model is required toward advanced tactile perception tasks such as schematically shown in Figure 2D.

One of the potential application of this technology could be in an industrial task such as fruit or object sorting, where the bio-mimicking neural networks in the skin of robotic hands could classify and held objects based on the physical parameters such as pressure, temperature etc. as well as optical parameters from special optical sensors from tactile e-skin (Dahiya and Valle, 2013; Dahiya et al., 2015).

## Conclusions

A novel υ-NWFET based approach for realizing hardware neural networks has been presented and validated through device, circuit and system-level modeling and simulation. Two different approaches, STM and LTM, have been simulated to implement the memory or neuroplasticity. Fabrication of a υ-NWFET has been carried out with a Si-NW as the channel material. The I-V characteristics of the υ-NWFET demonstrates the neuronal function of the device with synaptic weights modulating the output current. For example, for a given drain (V_DS_ = 4 V) and gate voltage (V_GS_ = 6 V), the drain current at output was reduced by ~54 times with a gate weight of 5/14 as compared to 2/14. The proposed structure is a step toward realizing flexible power-efficient bio-inspired neural sensing and circuit architectures as a backplane for tactile e-skin in robotics or prosthetics. To this end, the system model interfaced with a flexible and transparent touch sensitive e-skin (having 6 × 6 tactile elements) and tested in real-time demonstrates the working of the proposed approach. Up to 1% change in the weights of the system model lead to misclassification of the data set by <0.06% which shows the robustness of NN for tactile sensing application. In principle, the approach could be adapted for spiking neural networks and further exploration in that direction should be extremely interesting. Multilayer or deep learning hardware neural networks could be used for further additional sparse coding to enable advanced tactile perception tasks such as schematically shown in Figure 2D. Future work will include large area fabrication of the proposed e-skin system in a flexible form factor and its subsequent testing.

## Author contributions

WT and RD conceptualized the idea. WT, FL, and VV contributed to the simulation. WT, CG, and DS contributed to the fabrication and analysis of device. All the authors were involved in the manuscript preparation. RD provided overall supervision of this research.

### Conflict of interest statement

The authors declare that the research was conducted in the absence of any commercial or financial relationships that could be construed as a potential conflict of interest.
