# Supplementary material for: Nanowire FET Based Neural Element for Robotic Tactile Sensing Skin
Source: Front Neurosci. 2017 Sep 20;11:501. doi: 10.3389/fnins.2017.00501 (PMC5611376; doi:10.3389/fnins.2017.00501)
Supplement: Supplementary file 2 [file Presentation1.PDF]

Supplementary Material

Nanowire FET based Neural Element for Robotic Tactile Sensing System

William Taube Navaraj[1], Carlos G. Nunez[1], Dhayalan Shakthivel[1], Vincenzo Vinciguerra[2], Fabrice Labeau[3], Duncan Gregory[4] and Ravinder Dahiya[1]*

[1]Bendable Electronics and Sensing Technologies group, School of Engineering, University of Glasgow, UK.

[2]ST Microelectronics, Italy.

[3]McGill University, Montreal, Canada.

[4]School of Chemistry, University of Glasgow, UK

***Correspondence:** Ravinder.Dahiya@glasgow.ac.uk

1 Estimation of approximate distribution of mechanoreceptors in various parts of human skin

Fig. 1 gives estimate of a typical distribution of mechanoreceptors (MR) in various parts of the human skin excluding the . The purpose is to give an estimate of mechanoreceptors on various regions for researchers working on biomimetic prosthetic and robotic skin. The number of MRs on the glabrous region of the hand was extensively studied by Johansson *et al.* (Johansson and Vallbo, 1979) based on histological, neurophysiological and statistical analysis. These MRs are composed of Slow Adapting (SA) I , SA II, Fast Adapting (FA) I, FA II receptors. The total glabrous hand area considered for this calculation is 194 cm^{-2}, which is a mid-estimate of the subjects studied (rounded-off to nearest integer). From this, using the relative surface area of various glabrous skin regions, the absolute area was calculated which are given in Fig. 1. The MR densities are estimated as 241 cm^{-2} for the fingertips, 81 cm^{-2} for the rest of finger area and 58 cm^{-2} for the palmar region. From the absolute area, based on the mechanoreceptor densities the number of mechanoreceptors are reported at different regions. In case of the distal phalanges, the total number of sensors have been reported, divided into the MRs at fingertip region as well as the rest of the distal phalange region. The total number of mechanoreceptors are ~18675 in an area of 194 cm^{-2}, out of which ~13350 are in finger regions while ~5325 are in palmar regions. The total number of mechanoreceptors on the thumb, index, middle, ring and little finger are ~3374, ~2821, ~2800, ~2388, ~1967, respectively.

For the rest of the region, the estimate was carried out indirectly from reported dermatological and psychophysical studies (Boniol et al., 2008), (Mancini et al., 2014), (Goldstein and Brockmole, 2016). A direct estimate based on a whole body histological study is currently not available. The hairy skin of the body has both fast-conducting (myelinated Aβ fiber) and slowly-conducting (unmyelinated CT fiber) tactile systems. The proportional area of each regions was estimated based on relative surface area of various regions in the body of adult (Boniol et al., 2008). The proportional area has been

considered same for the glabrous and dorsal region of the hand and feet. Spatial acuity gives an indirect estimate of the number of mechanoreceptors which was corrected with correction factor as per histological studies in the hand regions applied to whole of the body. Even though, this is not exact, this is to give an indirect estimate for the typical number of MRs required for whole body tactile sensing in humanoid robots and prosthetics. Table 1 summarizes the results. Overall, the estimate indicates that ~45k mechanoreceptors are distributed across ~1.5m^2 area of typical human skin.

**Region**	**Relative Area %**	**Absolute Area cm^2**	**Spatial Acuity cm**	**# of MRs**	**Reference**
Hand (Glabrous)	1.2	194	Various	18675	(Johansson and Vallbo, 1979; Boniol et al., 2008)
Hand (Dorsum)	1.2	194	1.0	301	(Boniol et al., 2008; Mancini et al., 2014)
Foot (Glabrous)	1.5	252	0.8	611	(Boniol et al., 2008; Mancini et al., 2014)
Foot (Dorsam)	1.5	252	1.8	121	(Boniol et al., 2008; Mancini et al., 2014)
Head	3.9	644	0.76	1728	(Boniol et al., 2008; Mancini et al., 2014)
Shoulders	1.9	314	2.2	101	(Boniol et al., 2008; Mancini et al., 2014)
Forearm	3.0	487	1.7	261	(Boniol et al., 2008; Mancini et al., 2014)
Hindarm	4.8	793	4.5	61	(Boniol et al., 2008; Goldstein and Brockmole, 2016)
Chest	12.8	2114	3.2	319	(Boniol et al., 2008; Goldstein and Brockmole, 2016)
Back	13.9	2295	1.3	1358	(Boniol et al., 2008; Mancini et al., 2014)
Abdomen	2.9	479	3.6	57	(Boniol et al., 2008; Goldstein and Brockmole, 2016)
Thigh	9.2	1511	2.3	443	(Boniol et al., 2008; Mancini et al., 2014)
Leg	5.6	925	2.8	183	(Boniol et al., 2008; Mancini et al., 2014)

References:

Boniol, M., Verriest, J.-P., Pedeux, R., and Doré, J.-F. (2008). Proportion of skin surface area of children and young adults from 2 to 18 years old. *Journal of Investigative Dermatology* 128(2)**,** 461-464.

Goldstein, E.B., and Brockmole, J. (2016). *Sensation and perception.* Cengage Learning.

Johansson, R.S., and Vallbo, Å.B. (1979). Tactile sensibility in the human hand: relative and absolute densities of four types of mechanoreceptive units in glabrous skin. *The Journal of physiology* 286(1)**,** 283-300.

Mancini, F., Bauleo, A., Cole, J., Lui, F., Porro, C.A., Haggard, P., et al. (2014). Whole-body mapping of spatial acuity for pain and touch. *Ann Neurol* 75(6)**,** 917-924.
