# Supplementary material for: Nanowire FET Based Neural Element for Robotic Tactile Sensing Skin
Source: Front Neurosci. 2017 Sep 20;11:501. doi: 10.3389/fnins.2017.00501 (PMC5611376; doi:10.3389/fnins.2017.00501)
Supplement: Supplementary file 3 [file Presentation2.PDF]

Supplementary Material

Nanowire FET based Neural Element for Robotic Tactile Sensing System

William Taube Navaraj[1], Carlos G. Nunez[1], Dhayalan Shakthivel[1], Vincenzo Vinciguerra[2], Fabrice Labeau[3], Duncan Gregory[4] and Ravinder Dahiya[1]*

[1]Bendable Electronics and Sensing Technologies group, School of Engineering, University of Glasgow, UK.

[2]ST Microelectronics, Italy.

[3]McGill University, Montreal, Canada.

[4]School of Chemistry, University of Glasgow, UK

***Correspondence:** Ravinder.Dahiya@glasgow.ac.uk

2 Parameters used for Device (Silvaco ATLAS) and Circuit Simulations (Multisim)

```
Block-iterative    carriers = 2


CONSTANTS:
 Boltzmann's constant  = 1.38066e-023 J/K
 Elementary charge     = 1.60219e-019 C
 Permitivity in vacuum = 8.85419e-014 F/cm
 Temperature           = 300 K
 Thermal voltage       = 0.025852 V

REGIONAL MATERIAL PARAMETERS:
 Region       :         1         2         3         4         5         5         5         6         6         6
 Material     :       SiO2      HfO2      HfO2   Silicon    Nickel    Nickel    Nickel    Nickel    Nickel    Nickel
 Type         :    insulator insulator insulator semicond.   metal     metal     metal     metal     metal     metal

  Band Parameters
 Epsilon      :       3.9      20.0      20.0      11.8
 Eg (eV)      :                                    1.08
```

```
Chi (eV)      :                            4.17
Nc (per cc)   :                        2.8e+019
Nv (per cc)   :                       1.04e+019
ni (per cc)   :                       1.45e+010

Bandgap narrowing parameters
bgn.e (eV)    :                           0.009
bgn.n (/cc)   :                          1e+017
bgn.c        :                             0.5
ubgn.b        :                        3.1e+012
ubgn.c        :                        3.9e-005
bgn.shnk.me   :                           0.321
bgn.shnk.mh   :                           0.346
bgn.shnk.eps  :                            11.7
bgn.shnk.ge   :                              12
bgn.shnk.gh   :                               4

Effective Richardson Constants
An**          :                             110
Ap**          :                              30

Incomplete Ionization Parameters
Gc            :                               2
Gv            :                               4
Ed (eV)       :                           0.044
Ea (eV)       :                           0.045

Recombination Parameters
taun0         :                          1e-007
taup0         :                          1e-007
etrap         :                               0
nsrhn         :                          5e+016
nsrhp         :                          5e+016
ksrhtn        :                          0.0025
ksrhtp        :                          0.0025
ksrhcn        :                          3e-013
ksrhcp        :                       1.18e-012
ksrhgn        :                            1.77
ksrhgp        :                            0.57
nsrhn         :                          5e+016
nsrhp         :                          5e+016
augn          :                        2.8e-031
augp          :                        9.9e-032
augkn         :                               0
augkp         :                               0
kaugcn        :                       1.83e-031
```

```
 kaugcp       :                                  2.78e-031
 kaugdn       :                                       1.18
 kaugdp       :                                       0.72
 aug.cnl      :                                   2.2e-031
 aug.cpl      :                                   9.2e-032
 aug.chi      :                                  1.66e-030
 hns.ae       :                                   6.7e-032
 hns.ah       :                                   7.2e-032
 hns.be       :                                  2.45e-031
 hns.bh       :                                   4.5e-033
 hns.ce       :                                  -2.2e-032
 hns.ch       :                                  2.63e-032
 hns.he       :                                       3.47
 hns.hh       :                                       8.26
 hns.n0e      :                                     1e+018
 hns.p0e      :                                     1e+018
 copt         :                                          0

 Band-to-band tunneling Parameters
 mass.tunnel  :                                       0.25
 me.tunnel    :                                      0.322
 mh.tunnel    :                                      0.549

 Thermal Velocities
 vn (cm/s)    :                                  1.08e+007
 vp (cm/s)    :                                   1.3e+007

 Saturation Velocities
 vsatn (cm/s) :                                  1.03e+007
 vsatp (cm/s) :                                  1.03e+007


REGIONAL MOBILITY MODEL SUMMARY:

   Model for Electrons:

      Concentration Dependent Mobility
      @ Temperature =     300 Kelvin
      Using built-in model (refer to manual).

      Parallel Field Dependent Mobility
        Using parallel field model.
          Using built-in Silicon model for Vsat.
          alpha = 2.4e+007
          theta = 0.8
          tnom = 600
```

```
        beta = 2


  Model for Holes:

    Concentration Dependent Mobility
    @ Temperature =     300 Kelvin
    Using built-in model (refer to manual).

    Parallel Field Dependent Mobility
      Using parallel field model.
        Using built-in Silicon model for Vsat.
        alpha = 2.4e+007
        theta = 0.8
        tnom = 600
        beta = 1
```

Key Parameters:

Rel. Permittivity of HfO_2	:	20.0
Work function of Ni (eV)	:	5.01
Fixed Oxide Charge Density (cm^{-2})	:	10^{11}
Si-NW with width (nm)	:	100
height (nm)	:	100
length (µm)	:	15
Channel Doping (cm^{-3})	:	10^{14}
Source/Drain Doping (cm^{-3})	:	10^{20}

References:

(2015)

Silvaco (2015). Silvaco ATLAS User Manual.
