# Supplementary material for: Nanowire FET Based Neural Element for Robotic Tactile Sensing Skin
Source: Front Neurosci. 2017 Sep 20;11:501. doi: 10.3389/fnins.2017.00501 (PMC5611376; doi:10.3389/fnins.2017.00501)
Supplement: Supplementary file 4 [file Presentation3.PDF]

Supplementary Material

Nanowire FET based Neural Element for Robotic Tactile Sensing System

William Taube Navaraj[1], Carlos G. Nunez[1], Dhayalan Shakthivel[1], Vincenzo Vinciguerra[2], Fabrice Labeau[3], Duncan Gregory[4] and Ravinder Dahiya[1]*

[1]Bendable Electronics and Sensing Technologies group, School of Engineering, University of Glasgow, UK.

[2]ST Microelectronics, Italy.

[3]McGill University, Montreal, Canada.

[4]School of Chemistry, University of Glasgow, UK

***Correspondence:** Ravinder.Dahiya@glasgow.ac.uk

3 Output of Sensory Decay Neuron in System Model implemented in SimBrain

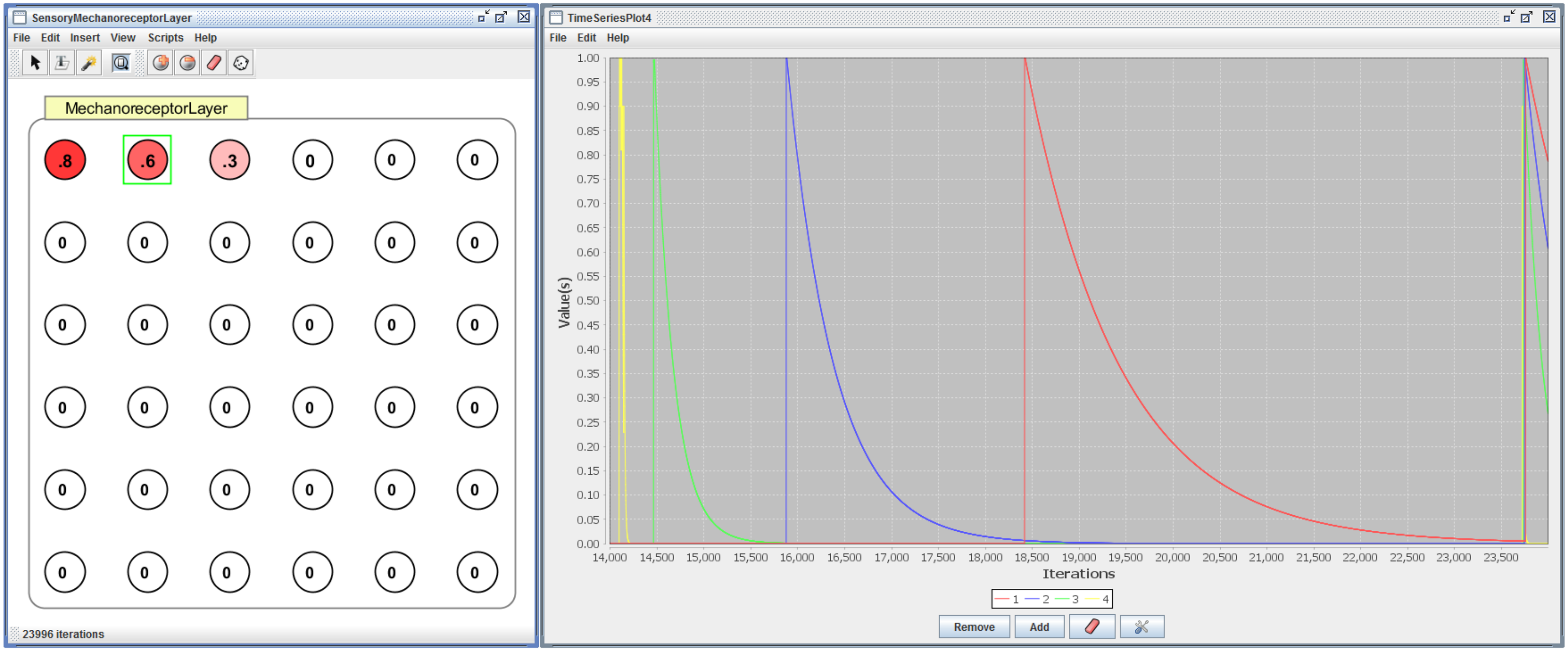

Fig. S3-1: Snapshot of the time series output of Sensory Decay Neuron in System Model implemented in SimBrain corresponding to decay constants Yellow(0.1), Green (0.005), Blue (0.002), Red (0.001),
